# Lead optimization of novel quinolone chalcone compounds by a structure–activity relationship (SAR) study to increase efficacy and metabolic stability

**DOI:** 10.1038/s41598-021-01058-z

**Published:** 2021-11-03

**Authors:** James Knockleby, Aïcha Dede Djigo, Indeewari Kalhari Lindamulage, Chandrabose Karthikeyan, Piyush Trivedi, Hoyun Lee

**Affiliations:** 1 Health Science North Research Institute, 56 Walford Road, Sudbury, ON P3E 2H3 Canada; 2 Department of Chemistry and Biochemistry, Laurentian University, 935 Ramsey Lake Road, Sudbury, ON P3E 2C6 Canada; 3 Departments of Medicine, University of Ottawa Medical School, Ottawa, ON K1H 5M8 Canada; 4 Department of Pharmacy, Indira Gandhi National Tribal University, Amarkantak, 484887 India; 5 Center of Innovation and Translational Research, Poona College of Pharmacy, Bharati Vidyapeeth Deemed University, Pune, 411 038 India

**Keywords:** Cancer, Cell biology, Chemical biology, Drug discovery, Oncology

## Abstract

Many agents targeting the colchicine binding site in tubulin have been developed as potential anticancer agents. However, none has successfully made it to the clinic, due mainly to dose limiting toxicities and the emergence of multi-drug resistance. Chalcones targeting tubulin have been proposed as a safe and effective alternative. We have shown previously that quinolone chalcones target tubulin and maintain potent anti-proliferative activity vis-à-vis colchicine, while also having high tolerability and low toxicity in mouse models of cancer and refractivity to multi-drug resistance mechanisms. To identify the most effective anticancer chalcone compound, we synthesized 17 quinolone–chalcone derivatives based on our previously published CTR-17 and CTR-20, and then carried out a structure–activity relationship study. We identified two compounds, CTR-21 [((E)-8-Methoxy-3-(3-(2-methoxyphenyl)-3-oxoprop-1-enyl) quinolin-2(1H)-one)] and CTR-32 [((E)-3-(3-(2-ethoxyphenyl)-3-oxoprop-1-enyl) quinolin-2(1H)-one)] as potential leads, which contain independent moieties that play a significant role in their enhanced activities. At the nM range, CTR-21 and CTR-32 effectively kill a panel of different cancer cells originated from a variety of different tissues including breast and skin. Both compounds also effectively kill multi-drug resistant cancer cells. Most importantly, CTR-21 and CTR-32 show a high degree of selectivity against cancer cells. In silico, both of them dock near the colchicine-binding site with similar energies. Whereas both CTR-21 and CTR-32 effectively prevents tubulin polymerization, leading to the cell cycle arrest at G2/M, CTR-21 has more favorable metabolic properties. Perhaps not surprisingly, the combination of CTR-21 and ABT-737, a Bcl-2 inhibitor, showed synergistic effect in killing cancer cells, since we previously found the “parental” CTR-20 also exhibited synergism. Taken together, CTR-21 can potentially be a highly effective and relatively safe anticancer drug.

## Introduction

Many different molecules have been developed over the last few years as potential anti-cancer agents^1–9^. One common target remains the microtubule cytoskeleton^10–12^. Microtubules, the polymers of alpha and beta tubulin proteins, are essential for a wide range of cellular functions including proliferation, intracellular trafficking, cell signaling, cell shape and migration, and even tumor angiogenesis. Drugs targeting microtubules have been shown to be very effective, with their potency even exceeding their anti-mitotic properties^13,14^. Microtubule-targeting agents (MTAs) have thus been widely used as chemotherapeutics for several decades and remain relevant in cancer therapy today, either administrated alone or in combination with other regimens. From colchicine, one of the oldest drugs, to more recent drugs such as paclitaxel and its analogues, MTAs interact with tubulin at different sites and through different mechanisms of actions (MOA)^15–17^.

Through studying the mechanisms of natural product MTAs, seven distinct drug binding sites have been identified as described below^1,7–11,15^ MTAs are broadly classified in two major groups: microtubule-stabilizing agents which promote microtubule polymerization while also inhibiting disassembly by binding to the tubulin polymer, and microtubule-destabilizing agents which bind to the tubulin dimers and block microtubule polymerization^10,11,17,18^. Two classes of compounds are considered microtubule-stabilizing agents: taxanes and laulimalide/peloruside. In contrast, there are five distinct binding sites on the tubulin subunit for compounds that can prevent microtubule polymerization. These molecule families are the vinca alkaloids, colchicine maytansine, pironetin^1,7,8,11,15^, and the recently described gatorbulin-1^10^. Although some of these molecules have been known for decades as anti-proliferative, they often suffer from some common drawbacks that limit their effectiveness in many cases, preventing their clinical use. These include acquired drug resistance and dose-limiting toxicities. The latter, in particular, prevents colchicine from being deployed as a cancer therapeutic^19,20^.

Our interest in finding novel safe and effective anticancer agents has focused on refining natural product compounds into lead candidates. One family of compounds that has shown great interest over the past few decades are chalcones. Chalcones are found in flavonoids in many different edible plants as secondary metabolites^21^. Chalcones contain a three carbon α, β unsaturated carbonyl core that links two phenyl rings. The phenyl rings offer sites of modification with a diversity of single and multiple additions in order to maximize effectiveness, increase stability, and enhance solubility and other desirable chemical properties. Chalcones that contain similar trimethoxyphenyl rings as colchicine show promise as anti-proliferative agents^1,8,22^, suggesting that methyoxy groups are likely important modifications for structure–activity studies.

Due to their simple chemistry which allows for an abundance of substitutions coupled with their known anti-proliferative activities, we synthesized 19 chalcone derivatives in total using the Claisen-Schimdt condensation and obtained the quinolone chalcone compounds (named CTRs) microtubule-destabilizing agents, two of which (CTR-17 and CTR-20) showed a selective and potent anti-tubulin activity and caused a prolonged mitotic arrest at the spindle assembly checkpoint (SAC), eventually lead to cell death^9^. In addition, both CTR-17 and CTR-20 did not show any notable side effects to mice^9^.

We sought to enhance their anti-growth/proliferation properties with a structure–activity relationship (SAR) approach to design and synthetize more effective candidates that would be more potent against cancer cells while sparing normal cells at low concentrations and maintaining selectivity. We identified that CTR-21 [((E)-8-Methoxy-3-(3-(2-methoxyphenyl)-3-oxoprop-1-enyl) quinolin-2(1H)-one)] is the most desirable anticancer agent among this series of chalcone compounds.

## Results

### Chemistry

**Figure 1:**
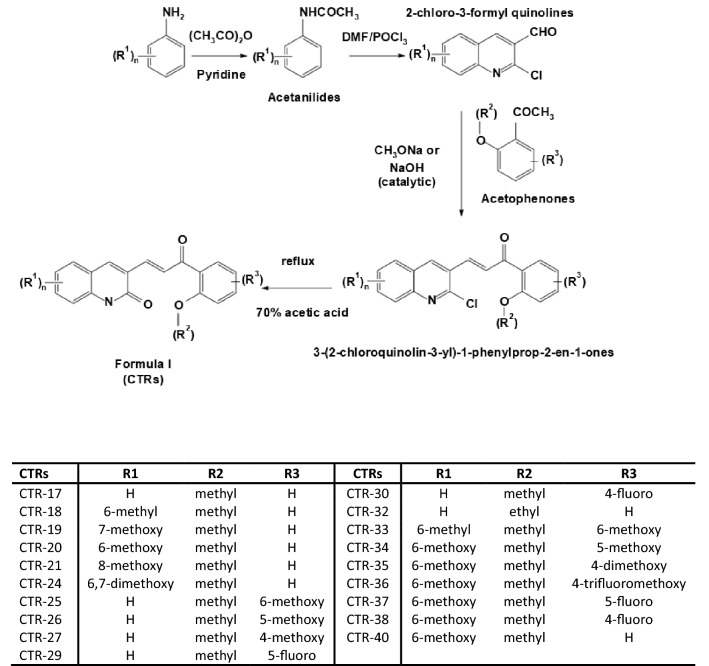
Synthesis Scheme and Structures for quinolone chalcones analogs.

The synthesis of the 19 quinolone chalcones were carried out using acetanilides commercially available or synthesized using standard protocols from anilines^23^ (Fig. 1). 2-chloroquinoline 3-carboxaldehydes were synthesized by treating acetanilides with DMF and POCl_3_ under Vilsmeier Haack conditions^24^. We then synthesized 3-(2-chloroquinolin-3-yl)-1-phenylprop-2-en-1-ones utilizing Claisen-Schmidt condensation of the 2-chloroquinoline-3-carbaldehydes under basic conditions (sodium methoxide or NaOH) with the appropriate acetophenones^25,26^ The 3-(2-chloroquinolin-3-yl)-1-phenylprop-2-en-1-ones were refluxed with aqueous glacial acetic acid to induce O-nucleophilic substitution at the 2-chloro group of the quinoline ring to derive the corresponding quinolone chalcones. Compounds were confirmed with a combination of their infrared (IR) spectroscopic ^1^H NMR and mass spectral data (see “Methods” section).

### CTR-21 and CTR-32 are the most effective anti-proliferative quinolone chalcones examined

We had initially determined that quinolone chalcones were promising anti-proliferative through a first round screening and identified CTR-17 and CTR-20 as favorable structures for anticancer activity^9^. CTR-17 and CTR-20 have been modelled to bind to the colchicine binding pocket on β–tubulin and cause a prolonged mitotic arrest at the spindle assembly checkpoint, eventually leading to apoptosis. We carried out a comparative SAR study to determine if we could further optimize the quinolone chalcone to maximize efficacy. The Sulforhodamine B (SRB) assay was used to measure the anti-proliferative/cell killing activities of CTRs, for which several different cell lines were use: the cervical cancer HeLa, the breast cancer cell lines MDA-MB231, MDA-MB468, MDA-MB231TaxR (an induced paclitaxel-resistant sub-cell line of the MDA-MB231^9^) and MCF7. In addition, a subset was tested against the melanoma cell lines MZ-MEL-3.1, Mel-SOE, UKRV-Mel-38, UKRV-Mel-17 and MDA-MB-435 (Table 1, Supplemental Table S1). CTR-21 and CTR-32 were highly potent with GI_50_ ranging from 5 to 91 nM. Notably, the ability for CTR-21 and CTR-32 to maintain effectiveness against MDA-MB231TaxR suggests that these two compounds remain refractive to multi-drug resistance mechanisms, as previously shown by CTR-20^9^ and is a common property of many colchicine site binders^1^ CTR-21 and CTR-32 were also highly effective against the NCI-60 panel of representative cancer cell lines (Supplemental Figs. S1–S8).

**Table 1:** GI_50_ values of quinolone chalcone analogs on breast cancer, melanoma and cervical cancer cells.

	Melanoma cell lines				Breast cancer cell lines					Cervical cancer
	MZ-Mel-3	Mel-SOE	UKRV-Mel-38	UKRV-Mel-17	MDA-MB435	MDA-MB231	MDA-MB231-TaxR	MDA-MB468	MCF7	HeLa
CTR17 (nM)	227 ± 30	786 ± 52	626 ± 154	504 ± 33	290 ± 66	657 ± 72	1299 ± 83	320 ± 26	485 ± 97	280 ± 78
CTR18 (nM)	239 ± 13	817 ± 34	746 ± 177	596 ± 162	307 ± 7	530 ± 119	1214 ± 179	304 ± 2	510 ± 38	443 ± 112
CTR19 (nM)	6832 ± 957	10,095 ± 64	3766 ± 206	11,166 ± 1660	4723 ± 2761	6506 ± 1388	7089 ± 1059	3538 ± 60	11,910 ± 564	6823 ± 1389
CTR20 (nM)	98 ± 21	338 ± 78	338 ± 33	283 ± 57	90 ± 10	216 ± 21	966 ± 163	408 ± 12	194 ± 21	124 ± 36
CTR21 (nM)	6 ± 1	27 ± 5	13 ± 3	9 ± 2	7 ± 2	17 ± 3	32 ± 8	29 ± 10	16 ± 5	8 ± 3
CTR32 (nM)	6 ± 2	33 ± 9	22 ± 3	18 ± 3	13 ± 5	20 ± 2	22 ± 4	26 ± 0	27 ± 6	11 ± 2
CTR40 (nM)	11 ± 0	53 ± 4	42 ± 11	28 ± 5	23 ± 0	30 ± 4	54 ± 13	77 ± 13	41 ± 5	19 ± 1
Tax (nM)^a^	58 ± 15	60 ± 16	6 ± 1	14 ± 4	4 ± 1	3 ± 1	131 ± 35	9 ± 3	6 ± 1	8 ± 1
Noco (nM)^a^	23 ± 2	51 ± 11	34 ± 3	39 ± 1	18 ± 2	35 ± 4	47 ± 11	48 ± 7	33 ± 7	25 ± 1

GI_50_ values were derived from a non-linear sigmoidal dose–response (variable slope) curve fitted by GraphPad Prism v.4.03 software.

^a^Tax and Noco denote paclitaxel and Nocodazole, respectively.

### SAR analysis reveals two separate moieties that synergistically increase anti-growth/proliferative effects

The diversity of chemical groups in our library allowed us to determine which moieties found on the two-ring structure might best enhance cytotoxicity (Fig. 1). CTR-17 shows the simplest design with an unsubstituted quinolone ring linked to 2-methoxy phenyl moiety through an ‘enone’ group and it displays an anti-proliferative activity in the medium range (GI_50_ = 464 nM; Table 1). The 2-methoxy group on the phenyl ring is critically important to the efficacy of the CTRs^27,28^. Introducing a 6-methyl group on the quinolone ring does not lead to any change in efficacy (CTR-18, GI_50_ = 499 nM; Fig. 1) with all 9 cell lines showing similar GI_50_ to that of CTR-17. The introduction of a 5-methoxy to CTR-17 (i.e., CTR-26) does not seem to have an effect either in terms of anti-growth/proliferative activity (GI_50_ = 443 nM), whereas CTR-29 (5-fluorophenyl) has a lower GI_50_ of 118 nM. In contrast, the addition of a 6-methoxy group on the phenyl ring (CTR-25, GI_50_ = 1.6 µM, Supplemental Table S1) leads to a fourfold increase in GI_50_. This suggests the possibility that the presence of a second methoxy group may induce steric hindrance or adverse interactions if a methoxy group is near the quinolone group. In contrast, a single methoxy group on the phenyl ring may not hinder the quinolone group, as the phenyl ring can rotate on the axis on its bond with the carbonyl of the enone group which may affect how the CTR binds to its target.

Examining the SAR of the methoxy group placement on the quinolone ring, we determined that if there is a methoxy group present, its position is vitally important to the cytotoxic activity of the compound. CTR-19, which has a 7-methoxy on the quinolone ring is the least effective analog out of the CTRs in terms of anti-proliferative activity with an average GI_50_ of 7.3 µM (Table 1), whereas the average GI_50_ of CTR-20 (6-methoxy) is 232 nM (Table 1) and CTR-21 (8-methoxy) is 16.4 nM (Table 1). The enhanced activity of CTR-20 is likely due to the presence of the methoxy on the C-6 since it is the only difference (i.e., methyl *vs* methoxy) from CTR-18 which shows much lower activity (Table 1). The activity of CTR-24 (6,7-dimethoxy quinolone) is between those of the 6-isomer and the 7-isomer (1.5 µM), which along with the lower toxicity potency of CTR-19 and CTR-23 (compared to CTR-17) suggesting that the 7- position should remain free for maximum efficacy, and that the 8-position is more favorable for cytotoxic activity than the 6-position.

The introduction of a 5-fluoro on CTR-20 (i.e., CTR-37) does not affect its activity contrary to what we observed between CTR-17 and CTR-29. However, when the fluorine group is added on the 4^th^ carbon (i.e., CTR-38), the average value of the GI_50_ drops to 98 nM which—while not making it as potent as CTR-21—still makes it one of the most active compounds. The addition of a methoxy group has differential effect on the potency of CTR-20 as well, depending on the position, as indicated by CTR-33 (6-methoxy, GI_50_ = 1.1 µM), CTR-34 (5-methoxy, GI_50_ = 648 nM), CTR-35 (4-methoxy, GI_50_ = 2 µM), and CTR-36 (4-trifluoromethoxy, GI_50_ = 805 nM). This evidence suggests that, although the 2-methoxy group on the phenyl ring is important for activity, we can enhance the activity of the compound by adding other moieties, but we may also impede activity by doing so.

Finally, substituting the 2-methoxyphenyl group for a 2-ethoxyphenyl improved the activity of the compounds. Indeed CTR-32 which, similarly to CTR-17, possesses no additional group on the quinolone but has a 2-ethoxy group on the phenyl ring (Fig. 1) and displays one of the lowest average GI_50_ values of the entire collection (20 nM; Table 1). Furthermore, another compound with a 2-ethoxygroup on the phenyl ring (in combination with a 6-methoxy on the quinolone ring), CTR-40 exhibited the third strongest activity (GI_50_ = 36 nM; Table 1). It is highly notable that replacing the 2-methoxy group of the phenyl group (CTR-17) by a 2-ethoxy group (CTR-32; Table 1) resulted in a 23-fold decrease in GI_50_. It is also remarkable that introducing an 8-methoxy on the quinolone group (CTR-21; Table 1) with keeping the 2-methoxyphenyl renders a 31-fold decrease. One concern of enhancing the activity of the CTR compounds is that toxicity may also increase towards normal cells. We previously showed that CTR-17 and CTR-20 are selective against malignant when compared their efficacies against non-malignant cells. Therefore, we next sought to determine the cytotoxicity of the most promising CTR compounds against primary melanocytes and peripheral blood mononuclear cells (PBMCs). We found that CTR-21 and CTR-32 are far less cytotoxic toward primary cells compared to cancer cell lines (Table 2). Comparing the selectivity index (SI) of the primary melanocytes versus the average GI_50_ of cancer cells, both CTR-21 and 32 have similar SIs (CTR-21: 157 fold; CTR-32: 158 fold; Table 2). There is a slightly smaller SI between cancer cells and PBMCs (CTR-21: 106 fold; CTR-32: 67 fold; Table 2), but still a substantial difference between normal and cancer cells. Taken together, these data indicate that we have identified two independent moieties on the CTR backbone that can enhance activity without sacrificing selectivity.

**Table 2:** GI_50_ values of quinolone chalcone analogs on primary skin and blood cells.

GI_50_	Primary skin cells			PBMC^a^		
	Melanocytes #1	Melanocytes #2	Selectivity Index	PBMC #1	PBMC #2	Selectivity Index
CTR-21 (nM)	2092 ± 804	3052 ± 960	157	1857 ± 336	1632 ± 317	106
CTR-32 (nM)	2631 ± 782	3627 ± 1083	158	1881 ± 147	787 ± 396	67

GI_50_ values were derived from a non-linear sigmoidal dose–response (variable slope) curve fitted by GraphPad Prism v.4.03 software.

^a^PBMC denotes peripheral blood mononuclear cells.

### CTR 21 reduces the steady state of microtubule polymerization by binding to β-tubulin

**Figure 2:**
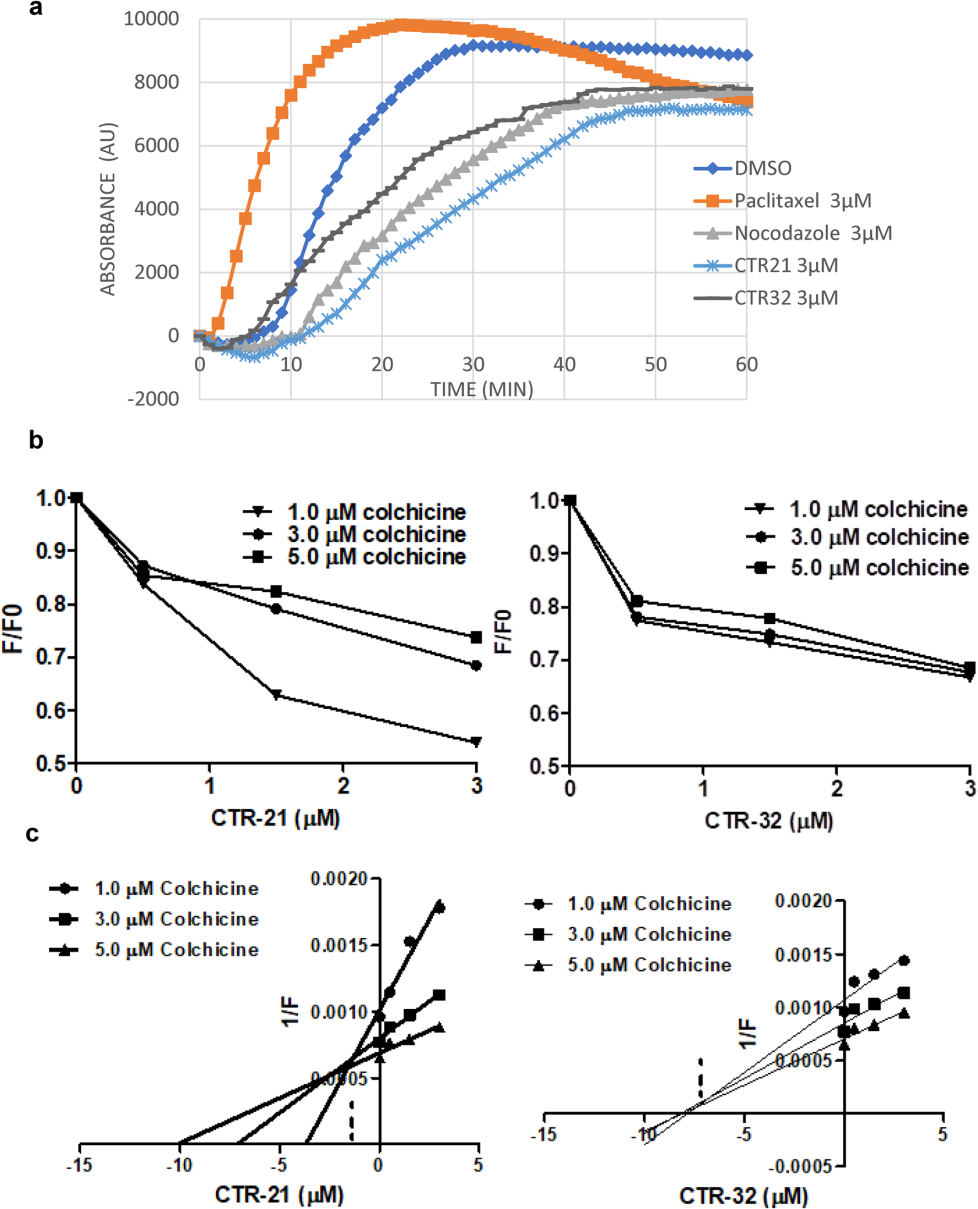
CTR-21 and CTR-32 inhibits microtubule polymerization and compete with colchicine through its binding to the pocket located at the colchicine- and gatorbulin-binding pockets in β-tubulin. (**a**) CTR-21 and CTR-32 inhibit microtubule polymerization in vitro. Spontaneous polymerization of 20 µM porcine tubulin was initiated with the addition of 1 mmol/L GTP at 37 °C and monitored every minute for 60 min in the presence of the indicated concentration of CTR-21, CTR-32, nocodazole, paclitaxel, or a DMSO vehicle control. To make the graph simpler, only 3 µM concentration is shown. The effects of higher doses are shown in Supplemental Fig. S9. (**b**) CTR-21 and CTR-32 inhibit the binding of colchicine to tubulin in a dose-dependent manner. The reduction of fluorescence of the tubulin-colchicine complex was measured in the presence of the indicated dose of CTR-21, CTR-32, or colchicine. Fluorescent intensity was compared to the fluorescence of the tubulin-colchicine complex in absence of CTR-21 or CTR-32 (F0). (**c**) CTR-21 can inhibit colchicine binding at a much lower dose than CTR-32. A modified Dixon plot was used to determine the inhibitory concentration of CTR-21 and CTR-32 (Ki).

We previously found that CTR-17 and CTR-20 inhibited microtubule polymerization as they compete with colchicine for binding to the colchicine-binding site^9^. Consistent with the notion that the CTRs play a role in disrupting microtubule dynamics, we next determined if the enhancement of efficacy seen in CTR-21 and CTR-32 were due to the disruption of microtubule dynamics in an in vitro tubulin polymerization assay (Fig. 2a; Supplemental Fig. S9). Compared to the control, the paclitaxel curve has a shorter nucleation phase, steeper growth phase, and reaches steady state quicker, as would be seen with a microtubule stabilizer. Nocodazole has a slightly lower V_max_ compared to the control (Supplemental Fig. S10) and a reduced steady-state acting as a microtubule destabilizer. Under these conditions, CTR-21 has a similar V_max_ to the control (Supplemental Fig. S10) yet a reduced steady-state, indicating that it is a tubulin destabilizer. CTR-32, however, does not affect the steady state dynamics or V_max_ of tubulin polymerization at 3 µM. However, since the concentration of tubulin is approximately 20 µM in this assay, and CTR-32 arrests the cells in G_2_/M (Supplemental Fig. S11), it is likely that CTR-32 affects tubulin dynamics in some fashion. Therefore, we next sought to determine the effects of higher concentrations of CTR-21 and 32, up to a stoichiometric 1:1 ratio of tubulin and CTR (Supplemental Fig. S9). We found that increasing levels of CTR-21 did not appreciably increase the inhibition of tubulin dynamics; this same phenomenon was observed with increasing the concentration of NZ. In contrast, when we increased the concentration of CTR-32, we found that even at 10 µM, it almost completely inhibited the formation of microtubule polymers, and was as effective at 15 and 20 µM (Supplemental Fig. S9). Therefore, at higher doses CTR-32 affects microtubule polymerization dynamics, and more dramatically than CTR-21 or NZ.

We previously showed through in vitro studies and in silico modeling that CTR-17 and CTR-20 bind to tubulin substantially overlapping the colchicine binding site, and inhibit tubulin polymerization^9^. Since there was a marked difference between the efficacy of CTR-20 compared to CTR-21 and CTR-32, we first sought to determine if CTR-21 and CTR-32 bind to the colchicine binding site. We used the intrinsic fluorescent nature of colchicine-tubulin complexes, as we carried out previously^9^, to determine if CTR-21 and CTR-32 could prevent colchicine binding to tubulin through competitive inhibition. When we added either CTR-21 or CTR-32 to the colchicine-tubulin complex, we observed a reduction in fluorescence in a dose -dependent manner (Fig. 2b).

When the data was plotted as a modified Dixon plot, we determined that the trendlines intersect above the X-axis for both CTR-32 and CTR-21 (Fig. 2c), indicating that they are competitive inhibitors. When we determined the Ki values, CTR-21 is about 4.9 times stronger inhibitor than CTR-32 (Ki values: CTR-21 1.47 µM; CTR-32 7.20 µM). Therefore, these data suggest that CTR-21 and CTR-32 bind tubulin at the colchicine binding site, but CTR-21 inhibits at a much lower concentration.

**Figure 3:**
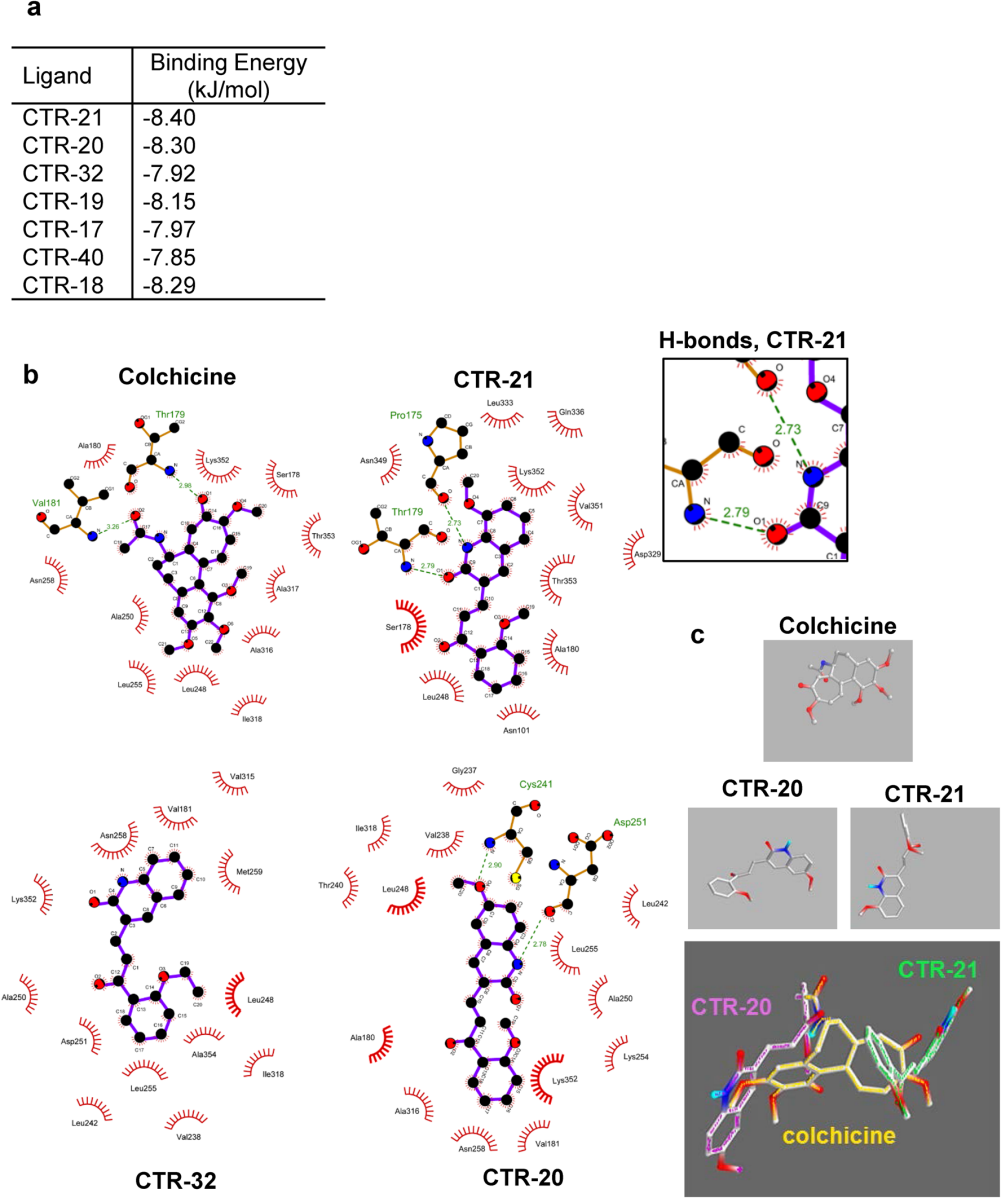
CTR-21 and CTR-32 may inhibit microtubule polymerization and compete with colchicine through its binding to the pocket located at the colchicine- and gatorbulin-binding pockets in β-tubulin. (**a**) CTRs dock with similar energies with alpha/beta tubulin. Shown are the best binding energies of CTRs in the interface between alpha and beta tubulin (PDB:4O2B). (**b**) CTR-21 is predicted to form hydrogen bonds with both α- and β-tubulin overlapping the colchicine-binding site. Two-dimensional representations of colchicine, CTR-20, CTR-21 and CTR-32 bound to interface between alpha and beta tubulin (PDB:4O2B) according to data obtained using the LigPlot + software^35^. Green lines indicate hydrogen bonds (with bond length in Ångstrom), red hemi-circles depict hydrophobic interactions. The color codes are as follows: oxygen, red; nitrogen, blue; sulfur, yellow; and carbon, black. The box is the enlarged portion of CTR-21 that forms hydrogen bonds with tubulin residues. (**c**) CTR-21 binds perpendicularly to colchicine. Three-dimensional representation of CTR-20 and CTR-21 bound in lowest energy poses from (**b**), in comparison with colchicine. Color codes are: oxygen, red; nitrogen, blue; and carbon, grey. In the bottom panel, the color codes of compound names and those of compound structures are the same.

We next sought to model the potential binding of these compounds in relation with each other. We found that the binding of CTR-21 and CTR-32 to the colchicine binding pocket did not have a notable difference in free energy when compared to CTR-20 or other CTRs (Fig. 3a), suggesting that the modifications did not adversely affect tubulin binding in general. However, the residues interacting with CTR-21 are different than the ones that interact with CTR-20 (Fig. 3b). CTR-21 potentially interacts with Pro175 of β-tubulin, a part of the recently identified gatorbulin binding site^10^. This site substantially overlaps the colchicine binding site and is the intramolecular equivalent of the vinblastine binding site on the intermolecular surface, but represents a potential novel and distinct binding site. This interaction is through hydrogen bonding with the quinolone ring nitrogen. CTR-21 interacts with Thr179 of α-tubulin part of the colchicine binding pocket, through hydrogen bonding with the carbonyl group on the quinolone ring. CTR-20 also interacts with a part of the colchicine-binding pocket, but the hydrogen bonding occurs on β-tubulin residues only, both between the quinolone methoxy group and the Cys241 residue on β-tubulin, and the quinolone nitrogen on Asp251 of β-tubulin. These in silico docking data suggest that CTR-21 interacts between the two subunits of the tubulin dimer, with some overlap with the colchicine binding site. Furthermore, the differences between the methoxy groups on CTR-20 and CTR-21 are evident when compared with colchicine in three-dimensional space (Fig. 3c). CTR-21 interacts in a perpendicular manner at the opposite end of the colchicine binding site when compared to CTR-20 (Fig. 3c). The carbonyls of both CTR-21 and colchicine form hydrogen bonds with the amino nitrogen of Thr179 in α-tubulin. Additionally, both compounds interact with Ala180 in a hydrophobic manner, CTR with the 2-methoxy phenyl group and colchicine with the acetamide group. However, the trimethoxy ring on colchicine points in a different direction compared to CTR-21 quinolone group, which interacts with Pro175 of the gatorbulin-binding site rather than further in the colchicine-binding domain. In contrast, CTR-20 fits into the colchicine site readily, nearly overlapping much of colchicine (Fig. 3c). These data suggests that the modifications of the quinolone ring are important for changing the way that the CTRs interact with the tubulin subunits in three-dimensional space. In particular, there are differences in how CTR-21 interacts with regard to hydrogen bonding and may explain why CTR-21 activity is much enhanced compared to CTR-20 and CTR-19, which contains a 7-methoxy group and arrests cells in G2/M, albeit at a much higher concentration (Supplementary Fig. S11).

The in silico docking may also give an indication as to why CTR-32 likely influences tubulin polymerization differently from that of CTR-21, in terms of competition at the colchicine binding site. In contrast to CTR-21, CTR-32 does not have any hydrogen bonding in the pocket. Its interactions are all hydrophobic, and the pose is different when compared to CTR-21 (Fig. 3b). The ethoxy group itself does not add any hydrogen binding potential to the compound, but does interact with β-tubulin though hydrophobic interactions. The docking suggests that CTR-32 has a different type of binding against β-tubulin than CTR-21. Nonetheless, the 2-ethoxy group on the phenyl group is important to the efficacy of the CTRs, since there is a large difference in average GI_50_ between CTR-17 and CTR-32. Therefore, both CTR-21 and CTR-32 influence tubulin dynamics, but as the in silico modeling suggests, the two molecules may take different poses which may explain the differential effects on polymerization at higher doses.

### CTR-21 is more metabolically stable than CTR-32

**Figure 4:**
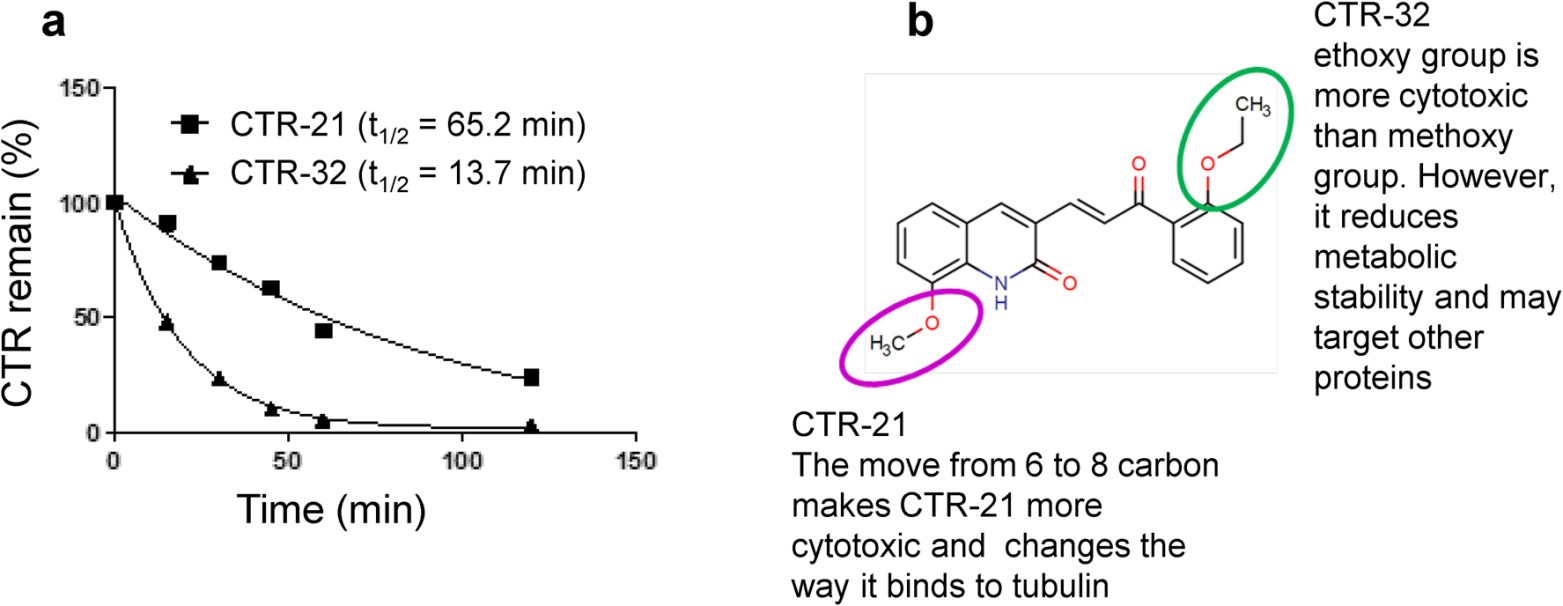
CTR-21 is the most active and stable one among this series. (**a**) CTR-21 is much more metabolically stable than CTR-32. 5 µM of CTR-21 or CTR-32 were incubated with human microsomes (20 mg/ml protein) in the presence of NADPH over the course of 120 min. The lysates were analyzed and % remaining CTR compounds were identified and estimated using HPLC–MS. (**b**) Summary of structure–activity relationship (SAR) based on activity and metabolic stability.

Toxicity and metabolic stability are other aspects of lead nomination that structural differences can affect. As part of pilot animal studies, we examined the toxicity of CTR-21 and CTR-32 in a mouse model and determined that there are no ill-effects observed in mice treated at 10 mg/kg, a dose that we had previously observed toxicity in paclitaxel-treated mice^9^ (Supplemental Fig. S12; Supplemental Table S2). These data suggest that CTR-21 and CTR-32 are likely not very toxic to the host. We next determined the effects of the structural changes that we made in the CTRs on in vitro metabolism using a human microsome model of CYP450 metabolism. We found that CTR-21 is much more stable than CTR-32, with a half-life almost five times greater than the latter (Fig. 4a). This data suggests that the ethoxy group on CTR-32 is a metabolic liability (Fig. 4b). Therefore, although CTR-32 is effective at killing cancer cells, it appears that the 2-ethoxy group makes it less stable than the 2-methoxy group seen in CTR-21. As such, we carried CTR-21 forward into further mechanistic studies, because it is the most promising agent among this series of quinolone chalcones (Fig. 3a).

### CTR-21 arrests cells in G_2_/M, leading to apoptosis

**Figure 5:**
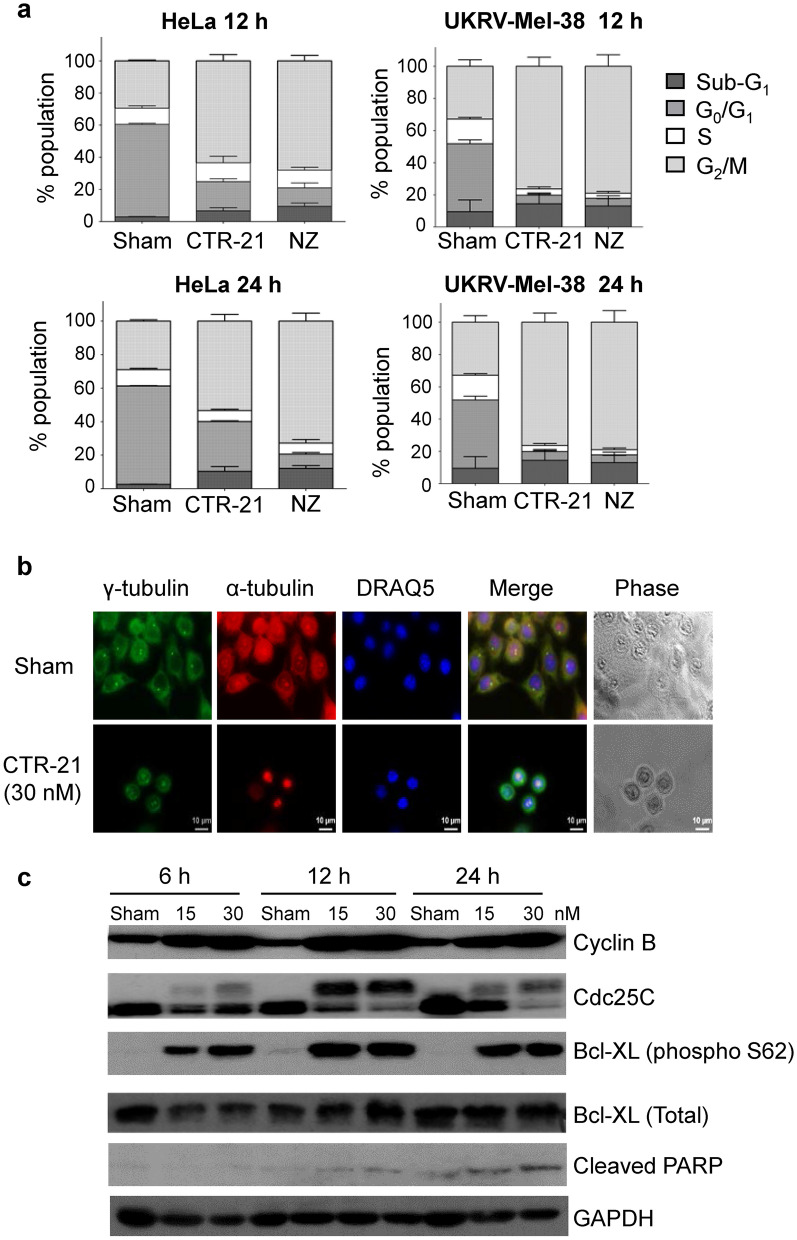
CTR-21 arrests cells at G2/M, eventually leading to cell death by apoptosis. (**a**) Effects of CTR-21 on the cell cycle progression of HeLa and E112 cells. Cells were treated with three times of GI_50_ concentrations of CTR-21 or nocodazole for 12 h and 24 h, stained with propidium iodide (PI), and then analysed cell cycle positions using flow cytometry. (**b**) Cell morphology of CTR-21 arrested cells. HeLa cells were treated with 30 nM of CTR-21 for 12 h, fixed with methanol and immunostained with α-tubulin or γ-tubulin, and counterstained with DAPI. Bar = 10 µM. (**c**) CTR-21 promotes PARP cleavage. Data from Western blotting with whole cell extracts of asynchronously growing population show that CTR-21 causes apoptosis, which is manifested by PARP cleavage. GAPDH was used as a loading control.

We anticipated that the changes made to enhance the efficacy of the CTR-21 backbone should not affect the mechanism of action since the compound could still bind to tubulin in silico and sought to determine if this was the case, using a combination of biochemical and cell biological approaches. We first examined the cell cycle effect of CTR-21 on HeLa and melanoma UKRV-Mel-38 cells (Fig. 5a). We found that HeLa cells arrest in G2/M by 12 h of post-treatment with 30 nM CTR-21, and maintain the arrest even after 24 h (Fig. 5a). We found that the majority of HeLa cells were rounded up with a short bi-polar spindle and condensed DNA by 12-h post-CTR-21 treatment, further indication of a G2/M arrest (Fig. 5b). Finally, Western blotting data also showed that cell populations arrest in G2/M until moving into apoptotic cell death (Fig. 5c). Asynchronous HeLa cells were treated with the indicated concentration of CTR-21 and monitored over the course of 24 h. By 6-h post-treatment, the levels of cyclin B and cdc25 phosphorylation (i.e., indicators of G2/M arrest) increased, and peaked at 12 h. Interestingly, the phosphorylation on the Ser62 residue of Bcl-XL is also substantially increased. By 24 h, we see an increase of PARP cleavage, indicating that apoptosis begins to occur 24-h post-CTR-21 treatment. Taken together from the flow cytometry and immunofluorescence studies, CTR-21 arrests cells in G2/M, much like the previously characterized analogs CTR-20 and CTR-17. This indicates that the structural changes made to CTR-21 have not substantially affected on the underlying molecular mechanism of cell death.

### CTR-21 shows synergistic effect when combined with either ABT-737 or paclitaxel

**Figure 6:**
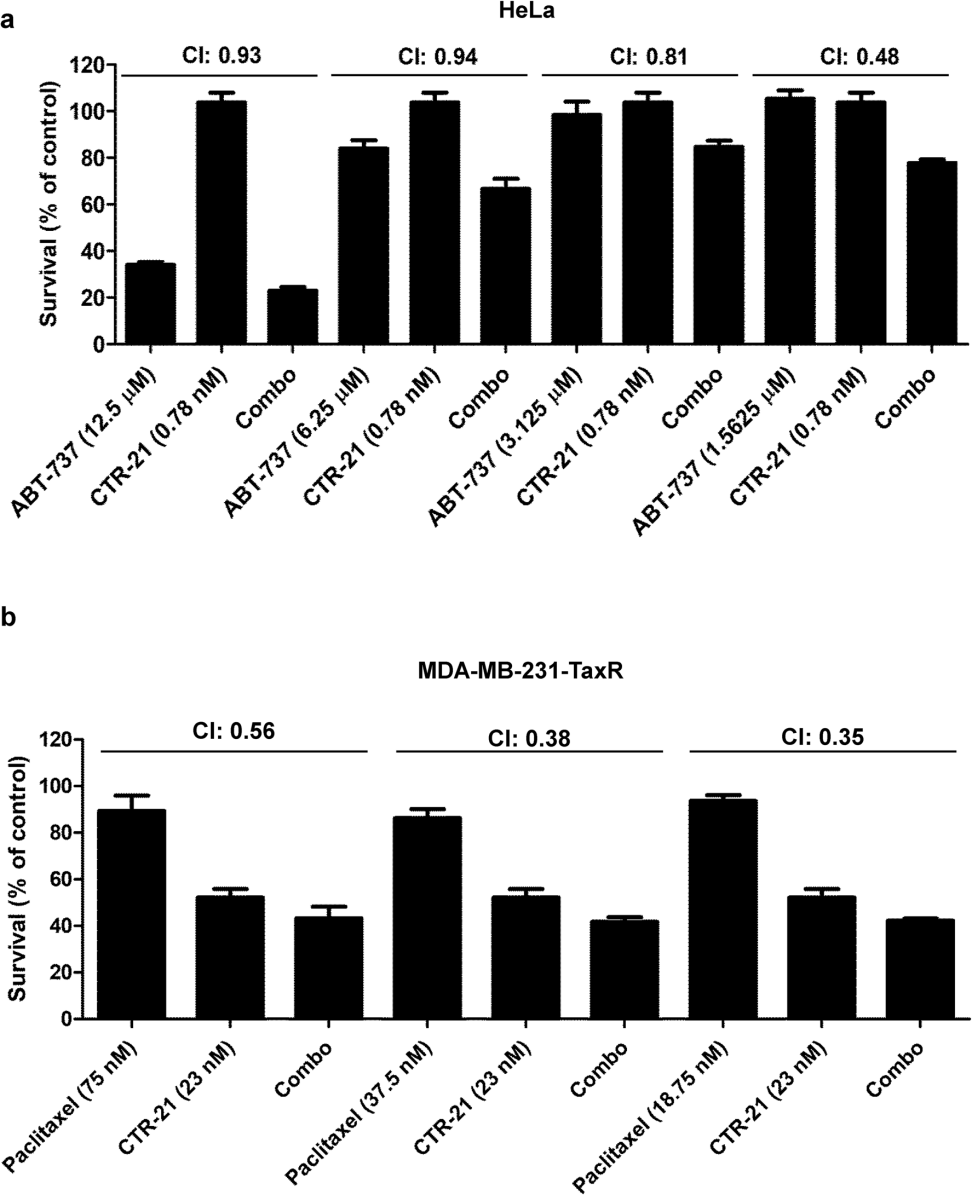
CTR-21 shows strong synergistic effects when combined with ABT-737 (Bcl-XL inhibitor) or paclitaxel. (**a**) CTR-21 kills HeLa in a synergistic manner with ABT-737. Cells were treated with 0.78 nM CTR-21 and indicated concentrations of ABT-737. CI denotes combinatorial index, calculated according to described previously^19^. (**b**) CTR-21 effectively kills paclitaxel-resistant cells when combined with paclitaxel. The paclitaxel-resistant MDA-MB-231TaxR cells were treated with 23 nM of CTR-21 and indicated concentrations of paclitaxel.

We previously showed that CTR-20 was synergistic with the Bcl-XL inhibitor ABT-737 in HeLa cells and enhanced the activity of paclitaxel in multidrug-resistant cells^9^. ABT-737 is limited in clinical use due to its severe side effect such as thrombocytopenia at the effective dose^29^. We determined if the changes we made to CTR-21 for enhancing its activity affected its ability to be synergistic with the anticancer agents. The complementary index (CI) was less than 1.0 when HeLa cells were treated with normally non-effective doses of CTR-21 (0.78 nM) in combination with low doses of ABT-737, indicating that there is clear synergism of this combination^30^ (Fig. 5a). The synergistic effect was especially pronounced (CI, 0.48) where each compound alone has little effect on the survival of the cells (i.e., 1.56 µM ABT-737 and 0.78 nM CTR-21) (Fig. 6a). This data may open the possibility that the combination of these two agents can be used to treat cancers without causing side effects. We found that in MDA-MB-231TaxR cells, which are normally resistant to paclitaxel, the combinatorial effects of CTR-21 and paclitaxel showed greater synergism than the combination with ABT-737 (Fig. 6b). Even at doses where paclitaxel has little effect on the cell (18.75 nM), the addition of 23 nM of CTR-21 can synergistically enhance the cell death (CI, 0.35). These data indicate that the SAR-directed enhancements made to the CTR backbone to make the highly effective CTR-21 did not change the ability of CTRs to work synergistically with potential combinatorial partners.

## Discussion

In an effort to optimize the efficacy and stability of novel quinolone chalcones CTR-17 and CTR-20, we created a small pool of derivatives with different modifications to the quinolone and/or phenyl ring moieties. We then carried out SAR analysis to determine the most effective CTR to serve as a lead in developing a highly desirable anticancer agent. We found that the most effective changes were moving the 6-methoxy group to the 8^th^ position on the quinolone ring (CTR-21), and replacing the 2-methoxy with an ethoxy group (CTR-32).

Both CTR-21 and CTR-32 are predicted to bind near the chalcone binding site in the α/β-tubulin dimer. This prediction by an in silico study is consistent with the fact that the cell cycle progression is effectively arrested at G2/M in the presence of either of the compounds, eventually leading to cell death by apoptosis. Although both CTR-21 and CTR-32 show similar effects on preventing tubulin polymerization and thus impeding normal microtubule functions, we consider that CTR-21 is more favorable as a potential drug since it is metabolically more stable than CTR-32.

Our data have clearly demonstrated that the methoxy group located at the 8th position on the quinolone ring is critically important for the efficacy enhancement of quinolone chalcones as CTR-21 with a methoxy group at that position is much more effective than the structural isomer CTR-19 (7-methoxy) or CTR-20 (6-methoxy). In silico docking studies provide some insights into why this may be the case. Comparing CTR-20 and CTR-21 suggests that CTR-21 forms two hydrogen bonds with tubulin dimers, interacting with the Pro175 of β-tubulin and Thr179 of α-tubulin residues. Thus, CTR-21 may impedes both α- and β-tubulin dynamic and functions. In contrast, CTR-20 only interacts with β-tubulin. Furthermore, CTR-21 is modeled to interact with tubulin in a unique manner, similar to the recently described gatorbulin binding site^10^, including the Pro175 of β-tubulin. Thus, the simple positional change of the methoxy group allows for the compound to position itself differently and as a result greatly increases its effectiveness.

It is unclear at this point why CTR-21 and CTR-32 are more effective against malignant tumor cells than normal cells (Tables 1 and 2). Theoretically, a microtubule inhibitor would be more effective against rapidly dividing cells. However, the doubling time of the primary skin cells used in our study is not necessarily longer than many cell lines listed in Table 1. Therefore, the SI is not directly correlated with the length of cell cycle, suggesting that there is other factor(s) involved in the preferential cancer cell killing by CTR-21and CTR-32.

Although the new chemical library that we created includes only a small number of chemicals, our data from this study allow us to generalize the repositioning effects on the phenyl rings of the quinolone chalcone backbone. Firstly, the methoxy group on the phenyl ring is essential for the anti-proliferative activity of the entire CTR library. Secondly, the presence of a methoxy group on the quinolone ring also adds to the efficacy of CTRs. It is known that methoxy groups are present in colchicine, and are necessary for its tubulin binding activity^31^ and thus its anti-proliferative activity^32^. Our finding is consistent with this previous finding. In addition, we furthered the previous report by unraveling the positional effects of the methoxy group. For example, CTR-19, which has a 7-methoxy is much less effective than its structural isomer CTR-21 which has an 8-methoxy group. Interestingly, the presence of more than one methoxy group on the quinoline ring diminishes the activity of CTR.

In conclusion, the novel CTR-21 compound, which was synthesized by modifying a few side chains from previously reported CTR-17 and CTR-20, dramatically (~ 30 fold) enhances the potency. Importantly, CTR-21 is highly selective in killing cancer over normal cells (SI is > 100 fold). Furthermore, unlike gatorbulin-1^10^ and other drugs targeting colchicine-binding site^1,9^, CTR-21 overcomes multi-drug resistance of malignant tumor cells. Finally, our preliminary animal data suggest that the increase in efficacy of CTR-21 has not increased its toxicity to the host animals. Taken together, we anticipate that CTR-21 has many positive characteristics to move it forward into the next stage of drug development using preclinical models.

## Methods

All chemicals and solvents used were commercially available and were of reagent grade. Where measurements are listed, melting points were determined in open glass capillaries on a Veego digital melting point apparatus and were uncorrected. The infrared (IR) spectra of compounds were recorded on Schimadzu FT-IR 8400S infrared spectrophotometer using an ATR accessory. 1H NMR spectra were recorded on a Bruker Avance II 400 spectrometer, using DMSO-d6 as solvent and tetramethylsilane (TMS) as internal standard. Mass spectral analysis was carried out using Applied Biosystem QTRAP 3200 MS/MS or Waters Xevo G2-XS Quadrupole Time-of-Flight mass spectrometer system in ESI mode. Reactions were monitored by TLC using pre-coated silica gel aluminum plates (Kieselgel 60, 254, E. Merck, Germany); zones were detected visually under ultraviolet irradiation.

### General synthesis of 2 chloro-3-formyl quinolines^24^

Commercially available Acetanilide /substituted acetanilides (0.05 mol) were dissolved in 9.6 mL of dimethyl formamide (DMF, 0.125 mol), to which 32 mL of phosphorus oxychloride (0.35 mol) was added gradually at 0 °C. The reaction mixture was taken in a round bottom flask (RBF) equipped with a reflux condenser fitted with a drying tube and was heated for 4–16 h on oil bath at 75–80 °C. The solution was then cooled to room temperature and subsequently poured onto 100 mL of icy water. The precipitate formed was collected by filtration and recrystallized from ethyl acetate.

### General synthesis of 2-chloroquinolinyl chalcones^25,26^

A mixture of 2-chloro-3-formyl quinolines (1 mmol), the respective acetophenones (1 mmol) and a base (either sodium methoxide (catalytic) or sodium hydroxide (one pellet)) in methanol (4 mL) was stirred at room temperature for 6–24 h. The resulting precipitate was collected by filtration, washed with water, and recrystallized from DMF-H_2_O or EtOH-H_2_O.

### General synthesis of 3-(3-oxo-3-phenylprop-1-enyl)quinolin-2(1H)-ones of formula I (Fig. 1)

A suspension of the 3-(2-chloroquinolin-3-yl)-1-phenylprop-2-en-1-ones (0.001 mol) in 70% acetic acid (10 mL) was heated under reflux for 4–6 h. Upon completion of the reaction (as indicated by a single spot in a TLC), the reaction mixture was cooled to ambient temperature and the solid product precipitated out was filtered. The filtered product was washed with water, dried and recrystallized in methanol or DMF/water.

### (E)-3-(3-(2-methoxyphenyl)-3-oxoprop-1-enyl)quinolin-2(1H)-one (CTR-17)

Yield 81%; M.P. 256–258 °C.; FT-IR (KBr) υ (cm^−1^): 3153 (NH), 1656 (C=O), 1586, 1557 (C=C), 1240, 1020 (C–O–C); 1H NMR (400 MHz, DMSO-d6): (12.05 (s, 1H), 8.47 (s, 1H), 7.86 (d, J = 16.0 Hz, 1H), 7.73 (d, J = 7.9 Hz, 1H), 7.60–7.44 (m, 4H), 7.34 (d, J = 8.3 Hz, 1H), 7.26–7.19 (m, 2H), 7.08 (td, J = 7.4, 0.9 Hz, 1H), 3.87 (s, 3H); MS-API: [M + H] + 306.1 (calculated 305.1).

### (E)-3-(3-(2-methoxyphenyl)-3-oxoprop-1-enyl)-6-methylquinolin-2(1H)-one (CTR-18)

Yield 86%; M.P. 222–224 °C.; FT-IR (KBr) υ (cm^−1^): 3145 (NH), 1654 (C=O), 1584, 1558 (C=C), 1241, 1019 (C–O–C); 1H NMR (400 MHz, DMSO-d6): δ 11.88 (s, 1H), 8.15 (s, 1H), 7.89 (d, J = 15.9 Hz, 1H), 7.58 (d, J = 15.9 Hz, 1H), 7.50 (t, J = 7.5 Hz, 2H), 7.44 (s, 1H), 7.32 (d, J = 8.5 Hz, 1H), 7.26 (d, J = 8.4 Hz, 1H), 7.10 (d, J = 8.3 Hz, 1H), 7.04 (t, J = 7.4 Hz, 1H), 3.90 (s, 3H), 2.39 (s, 3H), MS-API: [M + H] + 320.1 (calculated 319.12).

### (E)-7-Methoxy-3-(3-(2-methoxyphenyl)-3-oxoprop-1-enyl) quinolin-2(1H)-one (CTR-19)

Yield 83%; M.P. 227–229 °C.; FT-IR (KBr) υ (cm^−1^): 3144 (NH), 1656 (C=O), 1559 (C=C), 1167, 1021 (C—O—C); 1H NMR (400 MHz, DMSO-d6): δ 11.96 (s, 1H), 8.40 (s, 1H), 7.85 (d, J = 16.0 Hz, 1H), 7.60–7.42 (m, 3H), 7.32–7.18 (m, 4H), 7.08 (t, J = 7.4 Hz, 1H), 3.87 (s, 3H), 3.81 (s, 3H); MS-API: [M + H] + 336.1 (calculated 335.12).

### (E)-6-Methoxy-3-(3-(2-methoxyphenyl)-3-oxoprop-1-enyl) quinolin-2(1H)-one (CTR-20)

Yield 83%; M.P. 227–229 °C.; FT-IR (KBr) υ (cm^−1^); 3155 (NH), 1652 (C=O), 1597, 1558 (C=C), 1164, 1022 (C–O–C); 1H NMR (400 MHz, DMSO-d6): δ 11.91 (s, 1H), 8.37 (s, 1H), 7.78 (d, J = 15.9 Hz, 1H), 7.64 (d, J = 8.8 Hz, 1H), 7.57–7.49 (m, 2H), 7.48–7.40 (m, 1H), 7.20 (d, J = 8.5 Hz, 1H), 7.07 (t, J = 7.7 Hz, 1H), 6.89–6.81 (m, 2H), 3.85 (s, 3H), 3.84 (s, 3H); MS-API: [M + H] + 336.1 (calculated 335.12).

### (E)-3-(3-(2,6-dimethoxyphenyl)-3-oxoprop-1-enyl)quinolin-2(1H)-one (CTR-25)

Yield 65%; M.P. 236–238 °C.; FT-IR (ATR) υ (cm^−1^): 3149 (NH), 1667 (C=O), 1591, 1558 (C=C), 1252, 1058 (C–O–C); 1H NMR (400 MHz, DMSO-d6): δ 12.00 (s, 1H), 8.41 (s, 1H), 7.66 (d, J = 8.0 Hz, 1H), 7.47–7.55 (m, 1H), 7.32–7.41 (m, 2H), 7.21–7.31 (m, 2H), 7.18 (t, J = 7.6 Hz, 1H), 6.73 (d, J = 8.5 Hz, 2H), 3.69 (s, 6H); MS-API: [M + H] + 336.2 (calculated 335.12).

### (E)-3-(3-(2-ethoxyphenyl)-3-oxoprop-1-enyl)quinolin-2(1H)-one (CTR-32)

Yield 77%; M.P. 199–201 °C.; FT-IR (ATR) υ (cm^−1^): 3128 (NH), 1651 (C=O), 1597, 1555 (C=C), 1169, 1023 (C–O–C); 1H NMR (400 MHz, DMSO-d6): δ 12.02 (s, 1H), 8.39 (s, 1H), 8.00 (d, J = 15.8 Hz, 1H), 7.68 (dd, J = 8.0, 1.3 Hz, 1H), 7.44–7.56 (m, 4H), 7.26–7.32 (m, 1H), 7.11–7.23 (m, 2H), 7.02 (td, J = 7.5, 1.0 Hz, 1H), 4.12 (q, J = 7.0 Hz, 2H), 1.31 (t, J = 6.9 Hz, 3H). MS-API: [M + H] + 320.2 (calculated 319.12).

### (E)-3-(3-(2-ethoxyphenyl)-3-oxoprop-1-enyl)-6-methoxyquinolin-2(1H)-one (CTR-40)

Yield 86%; M.P. 233–235 °C.; FT-IR (KBr) υ (cm^−1^): 3166 (NH), 1652 (C=O), 1598, 1560 (C=C), 1245, 1023 (C–O–C); 1H NMR (400 MHz, DMSO-d6): δ 11.94 (s, 1H), 8.33 (s, 1H), 8.00 (d, J = 15.8 Hz, 1H), 7.43–7.53 (m, 3H), 7.17–7.26 (m, 3H), 7.15 (d, J = 8.3 Hz, 1H), 7.02 (t, J = 7.5 Hz, 1H), 4.12 (q, J = 6.8 Hz, 2H), 3.77 (s, 3H), 1.31 (t, J = 7.0 Hz, 3H); MS-API: [M + H] + 350.2 (calculated 349.13).

### (E)-8-methoxy-3-(3-(2-methoxyphenyl)-3-oxoprop-1-enyl) quinolin-2(1H)-one (CTR-21)

Yield 71%. 1H NMR (400 MHz, DMSO-d6): 3.86 (s, 3H), 3.91 (s, 3H), 7.06 (t, 1H), 7.17 (t, 3H), 7.29 (d, 1H), 7.47 (d, 1H), 7.52 (m, 2H), 7.86 (d, 1H), 8.45 (s 1H), 11.16 (1H). MS-API: [M + H] + 336.1 (Calculated: 335.12).

### (E)-6,7-dimethoxy-3-(3-(2-methoxyphenyl)-3-oxoprop-1-enyl)quinolin-2(1H)-one (CTR-24)

1H NMR (400 MHz, DMSO-d6) δ 11.83 (s, 1H), 8.27 (s, 1H), 7.74 (d, J = 16.01 Hz, 1H), 7.38–7.56 (m, 3H), 7.14–7.19 (m, 2H), 7.03 (dt, J = 0.88, 7.44 Hz,1H), 6.83 (s, 1H), 3.82 (s, 3H), 3.81 (s, 3H), 3.77 (s, 3H).

### (E)-3-(3-(2,5-dimethoxyphenyl)-3-oxoprop-1-enyl)quinolin-2(1H)-one (CTR-26)

1H NMR (400 MHz, DMSO-d6) δ 12.00 (s, 1H), 8.42 (s, 1H), 7.83 (d, J = 15.76 Hz, 1H), 7.69 (d, J = 8.00 Hz, 1H), 7.45–7.57 (m, 2H), 7.30 (d, J = 8.25 Hz,1H), 7.19 (t, J = 7.50 Hz, 1H), 7.04–7.14 (m, 2H), 6.98 (d, J = 2.75 Hz, 1H), 3.78 (s, 3H), 3.72 (s, 3H).

### (E)-3-(3-(2,4-dimethoxyphenyl)-3-oxoprop-1-enyl)quinolin-2(1H)-one (CTR-27)

1H NMR (400 MHz, DMSO-d6) δ 11.98 (s, 1H), 8.38 (s, 1H), 7.99 (d, J = 16.01 Hz, 1H), 7.70 (d, J = 7.75 Hz, 1H), 7.49–7.58 (m, 3H), 7.30 (d, J = 8.25 Hz,1H), 7.19 (t, J = 7.50 Hz, 1H), 3.86 (s, 3H), 3.83 (s, 3H).

### (E)-3-(3-(5-fluoro-2-methoxyphenyl)-3-oxoprop-1-enyl)quinolin-2(1H)-one (CTR-29)

1H NMR (400 MHz, DMSO-d6) δ 12.02 (s, 1H), 8.44 (s, 1H), 7.82 (d, J = 16.01 Hz, 1H), 7.69 (d, J = 7.75 Hz, 1H), 7.47–7.59 (m, 2H), 7.37 (dt, J = 3.25,8.63 Hz, 1H), 7.30 (d, J = 8.25 Hz, 1H), 7.26 (dd, J = 3.25, 8.76 Hz, 1H), 7.17–7.22 (m, 2H), 3.29 (s, 3H).

### (E)-3-(3-(4-fluoro-2-methoxyphenyl)-3-oxo prop-1-enyl)quinolin-2(1H)-one (CTR-30)

1H NMR (400 MHz, DMSO-d6) δ 12.01 (s, 1H), 8.42 (s, 1H), 7.87 (d, J = 16.01 Hz, 1H), 7.69 (d, J = 7.75 Hz, 1H), 7.48–7.58 (m, 3H), 7.30 (d, J = 8.25 Hz,1H), 7.19 (t, J = 7.50 Hz, 1H), 7.09 (dd, J = 2.25, 11.51 Hz, 1H), 6.87 (dt, J = 2.50, 8.38 Hz, 1H), 3.86 (s, 3H).

### (E)-3-(3-(2,5-dimethoxyphenyl)-3-oxoprop-1-enyl)-6-methoxyquinolin-2(1H)-one (CTR-34)

1H NMR (400 MHz, DMSO-d6) δ 11.92 (s, 1H), 8.36 (s, 1H), 7.82 (d, J = 16.01 Hz, 1H), 7.50 (d, J = 16.01 Hz, 1H), 7.18–7.26 (m, 3H), 7.07–7.12 (m, 2H), 6.98 (d, J = 2.50 Hz, 1H), 3.78 (s, 3H), 3.77 (s, 3H), 3.72 (s, 3H).

### (E)-3-(3-(2,4-dimethoxyphenyl)-3-oxoprop-1-enyl)-6-methoxyquinolin-2(1H)-one (CTR-35)

1H NMR (400 MHz, DMSO-d6) δ 11.89 (s, 1H), 8.31 (s, 1H), 7.99 (d, J = 15.76 Hz, 1H), 7.57 (d, J = 2.75 Hz, 1H), 7.54 (d, J = 9.76 Hz, 1H), 7.16–7.26 (m,3H), 6.67 (d, J = 2.25 Hz, 1H), 6.62 (dd, J = 2.25, 8.51 Hz, 1H), 3.87 (s, 3H), 3.83 (s, 3H), 3.77 (s, 3H).

### (E)-6-methoxy-3-(3-(2-methoxy-4-(trifluoro methoxy)phenyl)-3-oxoprop-1-enyl)quinolin-(1H)-one (CTR-36)

1H NMR (400 MHz, DMSO-d6) δ 11.94 (s, 1H), 8.37 (s, 1H), 7.83 (d, J = 15.76 Hz, 1H), 7.58 (d, J = 8.50 Hz, 1H), 7.51 (d, J = 15.76 Hz, 1H), 7.15–7.28 (m, 4H), 7.04 (d, J = 8.50 Hz, 1H), 3.87 (s, 3H), 3.77 (s, 3H).

### (E)-3-(3-(5-fluoro-2-methoxyphenyl)-3-oxoprop-1-enyl)-6-methoxyquinolin-2(1H)-one (CTR-37)

1H NMR (400 MHz, DMSO-d6) δ 11.94 (s, 1H), 8.37 (s, 1H), 7.82 (d, J = 16.01 Hz, 1H), 7.50 (d, J = 16.01 Hz, 1H), 7.37 (dt, J = 3.25, 8.63 Hz, 1H), 7.23–7.29 (m, 2H), 7.17–7.22 (m, 3H), 3.82 (s, 3H), 3.77 (s, 3H).

### (E)-3-(3-(4-fluoro-2-methoxyphenyl)-3-oxoprop-1-enyl)-6-methoxyquinolin-2(1H)-one (CTR-38)

1H NMR (400 MHz, DMSO-d6) δ 11.91 (br. s., 1H), 8.35 (s, 1H), 7.86 (d, J = 16.01 Hz, 1H), 7.48–7.58 (m, 2H), 7.17–7.29 (m, 3H), 7.09 (dd, J = 2.50,11.51 Hz, 1H), 6.88 (dt, J = 2.38, 8.44 Hz, 1H), 3.86 (s, 3H), 3.77 (s, 3H).

### Cell lines

The human MDA-MB-231, MCF7, MDA-MB-468, RPMI-8226 and HeLa cell lines were purchased from ATCC and maintained in Dulbecco's Modified Eagle Medium (DMEM)—high glucose supplemented with 10% fetal bovine serum (FBS) and antibiotics/antimycotics. Cell line authentication was carried out by Genetica DNA Laboratories (Burlington, NC) using a short tandem repeat (STR) profiling method (March 2015; July 2015; September 2016). Melanoma cells E-055 (MZ-Mel-3), E-097 (Mel-SOE), E-112 (UKRV-Mel-38), E-157 (UKRV-Mel-17) were obtained from the European Searchable Tumour Line Database (ESTDAB) cell bank (a kind gift of Dr. Graham Pawelec, Tubingen University, Germany). MDA-MB-435 was purchased from the Division of Cancer Treatment and Diagnosis Tumor Repository. All melanoma cells were cultured in Roswell Park Memorial Institute (RPMI) medium supplemented with 10% FBS and P/S/antimycotics. Primary human epidermal melanocytes from normal adult abdominal skin were purchased from ATCC (cat # PCS-200–013) and Cell Applications (cat#104-05a). The melanocytes were cultured in the media supplied by the companies.

### Sulforhodamine B (SRB) assay

The SRB assay was used to assess drug-induced cytotoxicity and cell proliferation as previously described^2,33,34^ with a few modifications. Briefly, cells were plated 10,000 to 25,000 cells/cm^2^ in 96-well plates (100 µl medium) and allowed to adhere overnight. The cell counts were different for each cell line to ensure optimal cell growth for the duration of the assay. Eight different concentrations of compound solutions using 2–4 times serial dilutions (100 µl). Cells were then incubated for an additional 48 h and then fixed with 100 µl ice-cold 10% (w/v) trichloroacetic acid (TCA) (without removing the cell medium, final concentration, 3.3% of TCA) and stained with 0.4% SRB (w/v) in 1% acetic acid (v/v). The relative growth rate (%) was calculated for each of the compound concentrations according to the following formula: 100 * (T − T0)/(C − T0), in which T is the optical density (OD) after exposure to a certain concentration of the compound after 48 h, T0 is the OD at the start of drug exposure (time zero) and C is the control growth. The GI_50_ for each compound was obtained from a non-linear sigmoidal dose–response (variable slope) curve fitted by GraphPad Prism v.4.03 software.

### PBMCs isolation

Peripheral blood mononuclear cells from healthy donors were isolated by density gradient centrifugation using Ficoll solution as described below. Blood samples used were supplied by the Canadian Blood Services under the Institutional Research Ethics Board Project # 18–099, and no human participants or volunteers were involved directly in the current study.

Whole blood was gently layered over an equal volume of Ficoll and centrifuged at 800 × *g* for 20 min with no brake. The Buffy coat was carefully collected by pipetting in 50 mL tubes and topped off with PBS. The cell isolates were subsequently mixed by inversion and centrifuged at 300 × *g* for 12 min. The supernatant was then carefully aspirated without disturbing the cell pellet, and the PBMCs were resuspended in ammonium-chloride-potassium (ACK) lysis buffer. The tubes were left to stand for 15 min to allow for red blood cell lysis. PBS was again added to the cells and centrifuged at 300 × *g* for 12 min and pelleted PBMCs were resuspended in RPMI + 10% heat inactivated FBS. Following counting, cells were centrifuged again and cryopreserved with a freezing medium composed of 40% FBS, 10% DMSO and 50% culture medium. Cryovials were placed in a − 80 °C for 24 h prior to transfer to liquid nitrogen for long-term storage.

### PBMCs thawing

PBMC cryovials were transferred from liquid nitrogen and placed in a 37 °C water bath until almost completely thawed. 1 mL of pre-warmed medium was added drop-wise and the entire contents of the cryovial was transferred to a 15-mL tube. Pre-warmed medium was then added to bring the volume up to 10 mL. The PBMCs were centrifuged at 300 × *g* for 10 min, and the cell pellet was gently resuspended with 5 mL of warm medium. The tubes were subsequently placed in a 37 °C incubator in a 5° angled rack for 4 h. An additional 5 mL warm medium containing of 30 units/mL benzonase nuclease was then injected into the cell solution and left to stand at room temperature for 5 min before centrifugation for 10 min at 300 × *g*. The pelleted cells were resuspended in the medium with 100 units/mL of IL-2 (Cedarlane Laboratories, Burlington, ON, Canada) and a small aliquot was used for cell counting and viability staining. The cells were plated in 96-well plates at a density of 80,000–120,000 cellules/well/100 µL.

### PBMCs culture and MTS assay

Following a 48-h incubation period, eight different concentrations of compound solutions using two to four-fold serial dilutions were added to the cells (100 µl). Cells were incubated for an additional 48 h and cytotoxicity was established using the CellTiter 96 cytotoxicity assay (Promega). 40 μl of the tetrazolium dye was added to each well of the plate and then incubated for 2 h. OD was then read directly at 490 nm using the automated Biotek Synergy H4 plate reader. The GI_50_ was calculated similarly to the SRB.

### Cell cycle analysis by flow cytometry

Cell cycle progression was determined using flow cytometry. UKRV-Mel-38 and HeLa cells were plated onto 10 cm plate at a density of 0.5 million and treated them with or without the compounds the following day for 12, 24 or 48 h. Cells were then harvested and centrifuged at 1000 rpm for 5 min, washed in PBS and then fixed with ice-cold ethanol (70%) for at least 24 h at − 20 °C. The cells were centrifuged, re-suspended in PBS solution, followed by centrifugation. The cell pellet was then stained with 100 μg/mL propidium iodide (PI) and 100 μg/mL RNase A in distilled water for at least 1 h. DNA content was measured using a Beckmann Coulter Cytomics FC500 (Beckman Coulter, Fullerton, CA), and the proportion of cell populations in G0/G1, S, and G2/M phases of cell cycle was calculated on the basis of DNA distribution histograms using CXP software (Beckman Coulter, Fullerton, CA).

### Cell cycle analysis by immunofluorescence and western blots

Cell cycle analysis, including immunofluorescence and western blot analysis was carried out as described previously^9^. Full versions of the blots are found in Supplemental Fig. S12.

### Tubulin assays

Microtubule assembly was assessed using a Tubulin Polymerization assay kit (BK011P; Cytoskeleton Inc., Denver, CO). 20 µM of purified porcine tubulin proteins (> 99% purity) were suspended in G-PEM buffer containing 80 mM PIPES, 2 mM MgCl2, 0.5 mM EGTA, 1 mM GTP (pH 6.9) and 20% glycerol. Polymerization was started by incubating at 37 °C and followed by absorbance readings every 1 min at 340 nm for 1 h using a Synergy H4 plate reader. Colchicine binding site assay was carried out as previously^9^.

### Microsome-based assay

Microsome metabolism assay was carried out as per manufacturer protocol (ThermoFisher), with 5 µM of starting compound. Analytes were measured on a Waters Xevo G2-XS Quadrupole Time-of-Flight mass spectrometer. Half-life was calculated using exponential one phase decay (GraphPad Prism 5). Hydroxychloroquine was used as an internal standard (500 pg/µL).

### Animal work

All animal experiments, including animal handling, care, treatments and endpoint determination were reviewed and approved by the Animal Care Committee (ACC) at Laurentian University (Sudbury, Ontario, Canada). We hereby confirm that all animal-based experiments were performed in compliance with relevant guidelines and regulations, including the ARRIVE guidelines (http://www.nc3rs.org.uk/page.asp?id=1357). Weight measurement and AST/ALT experiments were carried out as before^9^.

## Supplementary Information

Supplementary Information 1.

Supplementary Information 2.

Supplementary Information 3.
