# Supplementary material for: Lead optimization of novel quinolone chalcone compounds by a structure–activity relationship (SAR) study to increase efficacy and metabolic stability
Source: Sci Rep. 2021 Nov 3;11:21576. doi: 10.1038/s41598-021-01058-z (PMC8566451; doi:10.1038/s41598-021-01058-z)
Supplement: Supplementary file 1 — Supplementary Information 1. [file 41598_2021_1058_MOESM1_ESM.pdf]

Supplemental Information

Lead optimization of novel quinolone chalcone compounds by a structure-activity relationship (SAR) study to increase efficacy and metabolic stability

James Knockleby, Aicha Djigo1, I. Kalhari Lindamulage, Chandrabose Karthikeyan, Piyush Trivedi, & Hoyun Lee

Supplementary Table S1. Anti-proliferation effects of CTR compounds on cancer cells as determined by SRB assays

CODE	IC_{50} [a,b]			
	MDA-MB231	MCF7	HeLa	RPMI-8226
CTR-21 (nM)	56.91±10.46	75.11±3.07	11.93±1.40	5.34±0.89
CTR-24 (μM)	2.02±0.01	1.54±0.36	1.34±0.20	1.04±0.13
CTR-25 (μM)	2.60±0.18	1.69±0.07	1.32±0.02	0.77±0.21
CTR-26 (μM)	0.96±0.15	0.32±0.01	0.29±0.02	0.20±0.03
CTR-27 (μM)	2.69±0.24	2.20±0.35	1.80±0.27	0.77±0.10
CTR-29 (μM)	0.21±0.02	0.10±0.01	0.09±0.02	0.07±0.01
CTR-30 (μM)	1.42±0.08	1.47±0.15	1.50±0.13	0.20±0.03
CTR-32 (nM)	44.22±4.21	46.36±3.61	12.88±0.35	6.29±1.43
CTR-33 (μM)	1.36±0.11	1.71±0.25	0.10±0.20	1.17±0.11
CTR-34 (μM)	0.84±0.08	0.85±0.06	0.64±0.03	0.26±0.02
CTR-35 (μM)	2.35±0.26	2.12±0.36	2.07±0.25	1.32±0.10
CTR-36 (μM)	0.73±0.06	1.23±0.08	1.11±0.13	0.15±0.02
CTR-37 (μM)	0.39±0.09	0.32±0.04	0.14±0.03	0.10±0.03
CTR-38 (μM)	0.15±0.01	0.14±0.02	0.06±0.01	0.04±0.01
CTR-40 (μM)	0.13±0.02	0.09±0.01	0.04±0.00	0.05±0.02

[a] IC_{50} values were calculated from Sigmoidal dose response curves (variable slope), which were generated with GraphPad Prism V. 4.02 (GraphPad Software Inc.).
[b] Values are the mean value of triplicates of at least two independent experiments.

Supplemental Table S2. Summary of AST, ALT and AST/ALT values in CTR-21 and CTR-32.

Group	Sex*	Dose (mg/kg)	AST(U/L)	ALT(U/L)	AST/ALT
CTR-21	Female	1	249.47±77.12	118.03±25.82	2.12
		5	265.03±97.76	142.17±45.44	1.86
		10	319.215±101.93	142.98±45.61	2.23
	Male	1	264.42±91.94	128.49±60.78	2.06
		5	228.82±42.23	142.71±58.30	1.60
		10	242.62±102.65	123.93±49.96	1.96
Vehicle control	Female	0	279.65±84.23	116.02±33.99	2.41
	Male	0	312.39±100.53	103.28±27.46	3.02
CTR-32	Female	30	253.74±117.87	103.54±61.53	2.45
		50	268.16±136.88	87.81±25.42	3.05
	Male	30	288.49±184.97	110.30±44.56	2.62
		50	217.49±39.91	96.57±45.23	2.25
Vehicle control	Female	0	263.40±158.03	82.65±26.01	3.19
	Male	0	251.08±69.12	97.91±26.30	2.56

* Mouse strain used was C57BL/6 purchased from Charles River Laboratories. Inoculation of compound and other animal methods were as described previously (*Cancer Res* 75, 4164; *Sci Report* 7, 10298).

Supplementary Figure legend

Supplementary Figures S1-S4. CTR-21 effectively prevent proliferation of a representative set of cancer cell lines (NCI-60 panel). Figure S1: 10 μM of CTR-21 was used against the panel of NCI-60 cancer cell lines including: six leukemia cell lines, nine non-small cell lung cancer cell lines, seven colorectal cancer cell lines, six CNS cancer cell lines, nine melanoma cell lines, seven ovarian cancer cell lines, seven renal cancer cell lines, two prostate cancer cell lines and six breast cancer cell lines, utilizing the sulforhodamine B (SRB) colorimetric assay. Figure S2-S4: Five dose data for CTR-21.

Supplementary Figures S5-S8. CTR-32 effectively prevents proliferation of a representative set of cancer cell lines (NCI-60 panel). Figure S5: 10 μM of CTR-32 was used against the panel of NCI-60 cancer cell lines including: six leukemia cell lines, nine non-small cell lung cancer cell lines, seven colorectal cancer cell lines, six CNS cancer cell lines, nine melanoma cell lines, seven ovarian cancer cell lines, seven renal cancer cell lines, two prostate cancer cell lines and six breast cancer cell lines, utilizing the sulforhodamine B (SRB) colorimetric assay. Figure S6-S8: Five dose data for CTR-32.

Supplementary Figure S9. CTR-21 and CTR-32 inhibit microtubule polymerization *in vitro*. Spontaneous polymerization of 20 μM porcine tubulin was initiated with the addition of 1 mmol/L GTP at 37°C and monitored every minute for 60 minutes in the presence of the indicated concentration of CTR-21, CTR-32, nocodazole, paclitaxel, or a DMSO vehicle control.

Supplementary Figure S10. CTRs do not change the V_{max} of tubulin polymerization.

Supplementary Figure S11. Cell cycle assay of HeLa (**a**) and UKRV-Mel-38 (**b**). Cells were treated with 3× GI_{50} concentrations of compounds indicated for 12 or 24 hours, followed by staining with propidium iodide (PI) and analysis of cell cycle progression by flow cytometry.

Supplemental Figure S12: CTR-21 and CTR-32 do not show any notable toxicity to mice. (**a**) Neither CTR-21 nor CTR-32 treatment notably affected the body weight of mice treated with CTR-21 or CTR-32. Mice (C57BL/6) were injected (i.p.) every three days with the indicated doses of CTR-21 or CTR-32, along with vehicle control. All mice gained weight, even at the highest doses. (**b**, **c**) CTR-21 and CTR-32 do not show notable liver toxicity up to the maximum doses used in this experiment (i.e., 10 mg and 50 mg for CTR-21 and CTR-32, respectively). There is no significant changes of AST and ALT between different groups, including vehicle controls.

Developmental Therapeutics Program<br>One Dose Mean Graph	NSC: D-795903 / 1	Conc: 1.00E-5 Molar	Test Date: Feb 21, 2017
	Experiment ID: 1702OS60		Report Date: May 08, 2017

Panel/Cell Line	Growth Percent
**Leukemia**	
CCRF-CEM	-4.86
HL-60(TB)	-10.50
K-562	5.33
MOLT-4	7.38
RPMI-8226	-14.53
SR	-1.87
**Non-Small Cell Lung Cancer**	
A549/ATCC	21.38
EKVX	7.34
HOP-62	-8.85
HOP-92	10.59
NCI-H226	-22.05
NCI-H23	-16.39
NCI-H322M	8.09
NCI-H460	-27.45
NCI-H522	-72.66
**Colon Cancer**	
COLO 205	-49.71
HCC-2998	-84.73
HCT-116	-15.02
HCT-15	-12.50
HT29	-39.08
KM12	-49.06
SW-620	-6.28
**CNS Cancer**	
SF-268	2.36
SF-295	14.07
SF-539	-10.05
SNB-19	-4.63
SNB-75	34.28
U251	-55.75
**Melanoma**	
LOX IMVI	-63.52
MALME-3M	-0.69
M14	9.63
MDA-MB-435	6.42
SK-MEL-2	19.97
SK-MEL-28	2.94
SK-MEL-5	-39.25
UACC-257	33.57
UACC-62	-29.11
**Ovarian Cancer**	
IGROV1	2.88
OVCAR-3	-9.01
OVCAR-4	15.43
OVCAR-5	13.12
OVCAR-8	-5.37
NCI/ADR-RES	3.01
SK-OV-3	16.34
**Renal Cancer**	
786-0	9.27
A498	-15.03
ACHN	4.00
RXF 393	-43.46
SN12C	-0.14
TK-10	3.12
UO-31	-48.59
**Prostate Cancer**	
PC-3	31.14
DU-145	16.53
**Breast Cancer**	
MCF7	13.36
MDA-MB-231/ATCC	-0.93
HS 578T	39.27
BT-549	-3.55
T-47D	11.32
MDA-MB-468	-43.87
Mean	-7.56
Delta	77.17
Range	124.00

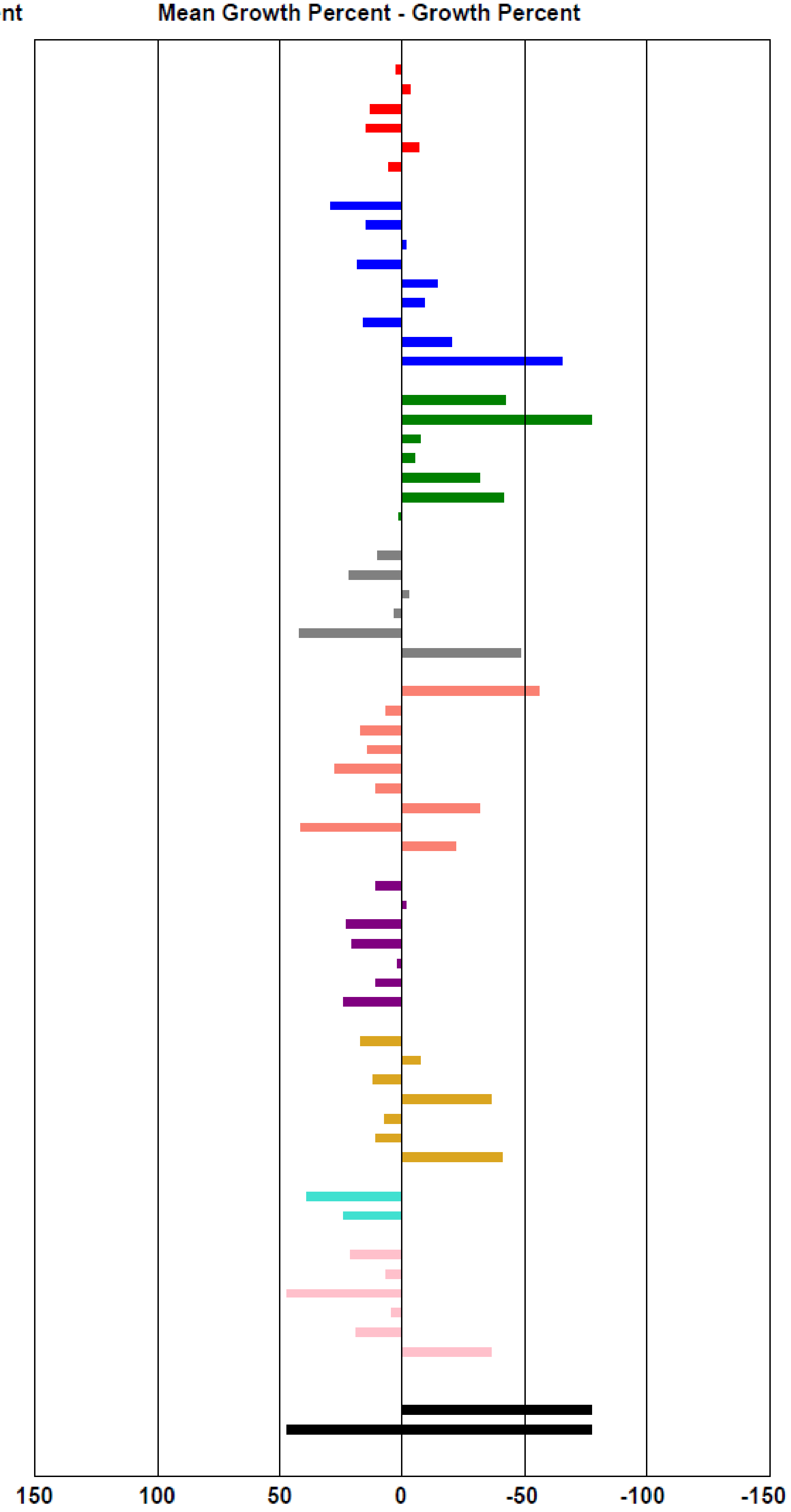

Fig. S1

National Cancer Institute Developmental Therapeutics Program<br>Dose Response Curves	NSC: D - 795903 / 1	SSPL: 0ZTS	EXP. ID: 1704NS87
	Report Date: May 08, 2017	Test Date: April 17, 2017	

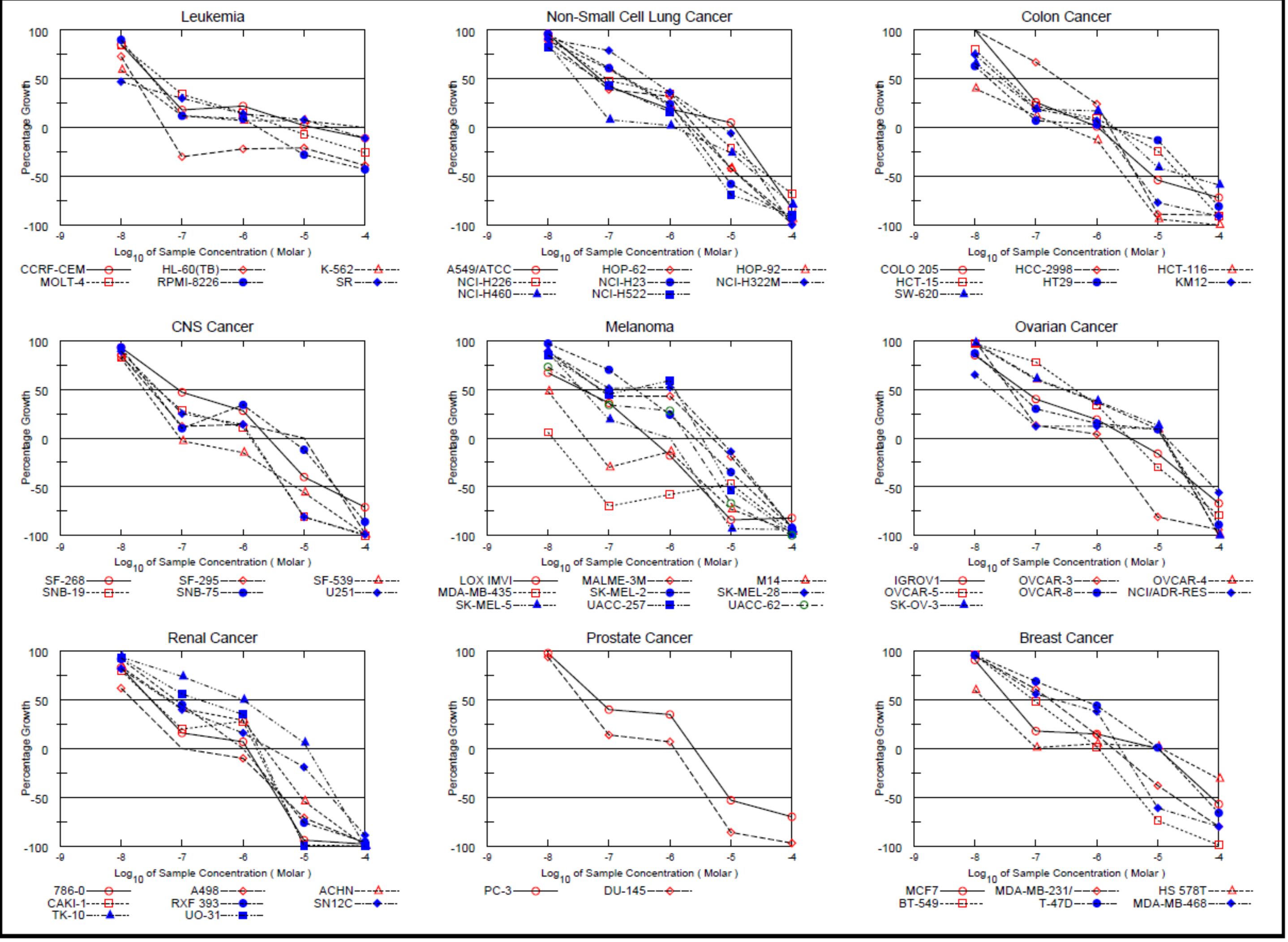

Fig. S2

National Cancer Institute Developmental Therapeutics Program
In-Vitro Testing Results

NSC : D - 795903 / 1	Experiment ID : 1704NS87	Test Type : 08	Units : Molar
Report Date : May 08, 2017	Test Date : April 17, 2017	QNS :	MC :
COMI : CTR 21	Stain Reagent : SRB Dual-Pass Related	SSPL : 0ZTS	

			Log10 Concentration													
	Time		Mean Optical Densities					Percent Growth								
Panel/Cell Line	Zero	Ctrl	-8.0	-7.0	-6.0	-5.0	-4.0	-8.0	-7.0	-6.0	-5.0	-4.0	GI50	TGI	LC50
**Leukemia**															
CCRF-CEM	0.434	1.384	1.242	0.603	0.643	0.454	0.386	85	18	22	2	-11	3.32E-8	1.43E-5	> 1.00E-4
HL-60(TB)	0.750	1.867	1.570	0.525	0.584	0.591	0.456	73	-30	-22	-21	-39	1.68E-8	5.13E-8	> 1.00E-4
K-562	0.269	1.586	1.045	0.432	0.366	0.357	0.267	59	12	7	7	.	1.55E-8	7.93E-5	> 1.00E-4
MOLT-4	0.735	2.182	1.961	1.220	0.953	0.682	0.543	85	34	15	-7	-26	4.77E-8	4.75E-6	> 1.00E-4
RPMI-8226	0.587	2.118	1.961	0.778	0.719	0.422	0.333	90	12	9	-28	-43	3.27E-8	1.72E-6	> 1.00E-4
SR	0.441	1.333	0.859	0.708	0.566	0.509	0.395	47	30	14	8	-11	< 1.00E-8	2.63E-5	> 1.00E-4
**Non-Small Cell Lung Cancer**															
A549/ATCC	0.361	1.574	1.525	0.873	0.594	0.419	0.062	96	42	19	5	-83	7.16E-8	1.13E-5	4.21E-5
HOP-62	0.745	2.019	1.882	1.244	1.151	0.430	0.007	89	39	32	-42	-99	6.07E-8	2.69E-6	1.37E-5
HOP-92	1.114	1.611	1.559	1.412	1.224	0.649	0.067	89	60	22	-42	-94	1.82E-7	2.21E-6	1.44E-5
NCI-H226	0.841	1.812	1.739	1.309	1.178	0.661	0.266	93	48	35	-21	-68	9.09E-8	4.15E-6	4.06E-5
NCI-H23	0.511	1.811	1.764	1.301	0.819	0.215	0.036	96	61	24	-58	-93	1.95E-7	1.95E-6	7.98E-6
NCI-H322M	0.874	2.013	1.915	1.770	1.290	0.818	-0.020	91	79	36	-6	-100	4.78E-7	7.09E-6	2.92E-5
NCI-H460	0.221	2.147	1.855	0.385	0.260	0.164	0.047	85	8	2	-26	-79	2.86E-8	1.18E-6	2.85E-5
NCI-H522	0.892	2.064	1.858	1.396	1.076	0.280	0.089	82	43	16	-69	-90	6.63E-8	1.54E-6	6.02E-6
**Colon Cancer**															
COLO 205	0.508	1.825	1.828	0.851	0.515	0.234	0.144	100	26	1	-54	-72	4.76E-8	1.02E-6	8.43E-6
HCC-2998	0.694	2.320	2.339	1.788	1.082	0.079	0.067	101	67	24	-89	-90	2.50E-7	1.63E-6	4.54E-6
HCT-116	0.195	1.417	0.678	0.347	0.171	0.011	-0.015	40	12	-13	-94	-100	< 1.00E-8	3.14E-7	2.87E-6
HCT-15	0.192	1.373	1.131	0.454	0.300	0.147	0.017	80	22	9	-24	-91	3.27E-8	1.90E-6	2.45E-5
HT29	0.230	1.350	0.938	0.304	0.258	0.200	0.043	63	7	3	-13	-81	1.71E-8	1.45E-6	3.48E-5
KM12	0.508	2.595	2.069	0.912	0.656	0.119	0.045	75	19	7	-77	-91	2.80E-8	1.21E-6	4.80E-6
SW-620	0.265	1.762	1.257	0.544	0.513	0.157	0.108	66	19	17	-41	-59	2.19E-8	1.94E-6	3.09E-5
**CNS Cancer**															
SF-268	0.537	1.918	1.827	1.192	0.927	0.320	0.158	93	47	28	-40	-71	8.78E-8	2.58E-6	2.07E-5
SF-295	0.604	2.296	2.037	0.806	0.847	0.609	0.013	85	12	14	.	-98	3.00E-8	1.01E-5	3.25E-5
SF-539	1.012	2.615	2.323	0.982	0.858	0.450	0.006	82	-3	-15	-56	-99	2.37E-8	9.23E-8	7.27E-6
SNB-19	0.445	1.798	1.573	0.831	0.593	0.084	-0.009	83	28	11	-81	-100	4.05E-8	1.31E-6	4.58E-6
SNB-75	0.886	1.716	1.656	0.970	1.165	0.776	0.123	93	10	34	-12	-86	3.29E-8	5.38E-6	3.23E-5
U251	0.334	1.474	1.347	0.615	0.496	0.064	0.002	89	25	14	-81	-99	4.03E-8	1.41E-6	4.74E-6
**Melanoma**															
LOX IMVI	0.333	2.312	1.666	1.046	0.273	0.053	0.062	67	36	-18	-84	-82	3.57E-8	4.64E-7	3.04E-6
MALME-3M	0.572	1.196	1.130	0.843	0.840	0.466	0.048	89	43	43	-19	-92	7.18E-8	5.00E-6	2.70E-5
M14	0.420	1.512	0.944	0.292	0.360	0.115	0.018	48	-30	-14	-73	-96	< 1.00E-8	4.09E-8	4.09E-6
MDA-MB-435	0.592	2.405	0.699	0.179	0.247	0.317	0.022	6	-70	-58	-47	-96	< 1.00E-8	1.20E-8	.
SK-MEL-2	1.031	2.081	2.051	1.767	1.279	0.667	0.078	97	70	24	-35	-92	2.70E-7	2.51E-6	1.81E-5
SK-MEL-28	0.753	2.248	2.090	1.515	1.532	0.651	0.064	89	51	52	-14	-92	1.08E-6	6.21E-6	2.93E-5
SK-MEL-5	0.632	2.939	2.656	1.073	0.636	0.047	0.037	88	19	.	-93	-94	3.55E-8	1.00E-6	3.48E-6
UACC-257	1.062	1.992	1.855	1.480	1.614	0.503	0.025	85	45	59	-53	-98	.	3.39E-6	9.46E-6
UACC-62	0.631	2.445	1.953	1.246	1.135	0.207	-0.008	73	34	28	-67	-100	3.86E-8	1.96E-6	6.59E-6
**Ovarian Cancer**															
IGROV1	0.375	1.737	1.536	0.923	0.636	0.316	0.125	85	40	19	-16	-67	6.07E-8	3.52E-6	4.70E-5
OVCAR-3	0.417	1.450	1.459	0.552	0.462	0.078	0.025	101	13	4	-81	-94	3.79E-8	1.12E-6	4.31E-6
OVCAR-4	0.826	1.664	1.640	1.333	1.137	0.899	0.012	97	60	37	9	-99	2.81E-7	1.20E-5	3.52E-5
OVCAR-5	0.581	1.340	1.314	1.170	0.840	0.406	0.120	97	78	34	-30	-79	4.31E-7	3.39E-6	2.53E-5
OVCAR-8	0.581	2.200	1.982	1.064	0.819	0.729	0.065	87	30	15	9	-89	4.40E-8	1.24E-5	4.01E-5
NCI/ADR-RES	0.487	1.677	1.265	0.632	0.626	0.590	0.213	65	12	12	9	-56	1.94E-8	1.36E-5	7.98E-5
SK-OV-3	0.894	1.865	1.842	1.484	1.262	1.023	-0.026	98	61	38	13	-100	2.95E-7	1.31E-5	3.62E-5
**Renal Cancer**															
786-0	0.403	1.704	1.477	0.615	0.497	0.023	0.009	83	16	7	-94	-98	3.10E-8	1.18E-6	3.66E-6
A498	1.422	2.138	1.869	1.410	1.276	0.412	0.049	62	.	-10	-71	-97	1.57E-8	9.70E-8	4.51E-6
ACHN	0.379	1.616	1.423	0.884	0.735	0.175	-0.026	84	41	29	-54	-100	6.14E-8	2.23E-6	8.96E-6
CAKI-1	0.767	2.725	2.338	1.152	1.322	0.009	-0.036	80	20	28	-99	-100	3.16E-8	1.67E-6	4.13E-6
RXF 393	0.685	1.324	1.271	0.974	0.679	0.163	0.030	92	45	.	-76	-96	7.86E-8	9.57E-7	4.48E-6
SN12C	0.391	1.688	1.448	0.905	0.596	0.316	0.044	82	40	16	-19	-89	5.65E-8	2.83E-6	2.77E-5
TK-10	0.865	1.691	1.645	1.477	1.279	0.911	-0.015	94	74	50	6	-100	1.01E-6	1.13E-5	3.36E-5
UO-31	0.608	2.046	1.942	1.419	1.110	0.002	-0.019	93	56	35	-100	-100	1.98E-7	1.82E-6	4.27E-6
**Prostate Cancer**															
PC-3	0.440	1.367	1.345	0.811	0.763	0.205	0.133	98	40	35	-53	-70	6.71E-8	2.48E-6	9.15E-6
DU-145	0.338	1.482	1.417	0.493	0.420	0.048	0.009	94	14	7	-86	-97	3.53E-8	1.19E-6	4.11E-6
**Breast Cancer**															
MCF7	0.346	2.183	2.009	0.684	0.616	0.350	0.150	91	18	15	.	-57	3.65E-8	1.01E-5	7.64E-5
MDA-MB-231/ATCC	0.547	1.431	1.386	1.085	0.674	0.337	0.108	95	61	14	-38	-80	1.71E-7	1.87E-6	1.89E-5
HS 578T	0.815	1.585	1.276	0.827	0.854	0.827	0.566	60	1	5	2	-31	1.47E-8	1.12E-5	> 1.00E-4
BT-549	1.139	1.947	1.916	1.526	1.153	0.296	0.015	96	48	2	-74	-99	9.02E-8	1.05E-6	4.82E-6
T-47D	0.703	1.578	1.542	1.307	1.086	0.711	0.237	96	69	44	1	-66	5.68E-7	1.03E-5	5.71E-5
MDA-MB-468	0.640	1.326	1.301	1.024	0.902	0.253	0.127	96	56	38	-61	-80	2.16E-7	2.44E-6	7.82E-6

Fig. S3

National Cancer Institute Developmental Therapeutics Program Mean Graphs	NSC : D - 795903/1	Units :Molar	SSPL :0ZTS	EXP. ID :1704NS87
	Report Date :May 08, 2017		Test Date :April 17, 2017	

Panel/Cell Line	Log_{10}GI50	Log_{10}TGI	Log_{10}LC50
**Leukemia**			
CCRF-CEM	-7.48	-4.84	> -4.00
HL-60(TB)	-7.77	-7.29	> -4.00
K-562	-7.81	-4.10	> -4.00
MOLT-4	-7.32	-5.32	> -4.00
RPMI-8226	-7.49	-5.77	> -4.00
SR	< -8.00	-4.58	> -4.00
**Non-Small Cell Lung Cancer**			
A549/ATCC	-7.15	-4.95	-4.38
HOP-62	-7.22	-5.57	-4.86
HOP-92	-6.74	-5.65	-4.84
NCI-H226	-7.04	-5.38	-4.39
NCI-H23	-6.71	-5.71	-5.10
NCI-H322M	-6.32	-5.15	-4.53
NCI-H460	-7.54	-5.93	-4.55
NCI-H522	-7.18	-5.81	-5.22
**Colon Cancer**			
COLO 205	-7.32	-5.99	-5.07
HCC-2998	-6.60	-5.79	-5.34
HCT-116	< -8.00	-6.50	-5.54
HCT-15	-7.48	-5.72	-4.61
HT29	-7.77	-5.84	-4.46
KM12	-7.55	-5.92	-5.32
SW-620	-7.66	-5.71	-4.51
**CNS Cancer**			
SF-268	-7.06	-5.59	-4.68
SF-295	-7.52	-5.00	-4.49
SF-539	-7.62	-7.03	-5.14
SNB-19	-7.39	-5.88	-5.34
SNB-75	-7.48	-5.27	-4.49
U251	-7.40	-5.85	-5.32
**Melanoma**			
LOX IMVI	-7.45	-6.33	-5.52
MALME-3M	-7.14	-5.30	-4.57
M14	< -8.00	-7.39	-5.39
MDA-MB-435	< -8.00	-7.92	
SK-MEL-2	-6.57	-5.60	-4.74
SK-MEL-28	-5.97	-5.21	-4.53
SK-MEL-5	-7.45	-6.00	-5.46
UACC-257		-5.47	-5.02
UACC-62	-7.41	-5.71	-5.18
**Ovarian Cancer**			
IGROV1	-7.22	-5.45	-4.33
OVCAR-3	-7.42	-5.95	-5.37
OVCAR-4	-6.55	-4.92	-4.45
OVCAR-5	-6.37	-5.47	-4.60
OVCAR-8	-7.36	-4.91	-4.40
NCI/ADR-RES	-7.71	-4.87	-4.10
SK-OV-3	-6.53	-4.88	-4.44
**Renal Cancer**			
786-0	-7.51	-5.93	-5.44
A498	-7.80	-7.01	-5.35
ACHN	-7.21	-5.65	-5.05
CAKI-1	-7.50	-5.78	-5.38
RXF 393	-7.10	-6.02	-5.35
SN12C	-7.25	-5.55	-4.56
TK-10	-6.00	-4.95	-4.47
UO-31	-6.70	-5.74	-5.37
**Prostate Cancer**			
PC-3	-7.17	-5.61	-5.04
DU-145	-7.45	-5.92	-5.39
**Breast Cancer**			
MCF7	-7.44	-5.00	-4.12
MDA-MB-231/ATCC	-6.77	-5.73	-4.72
HS 578T	-7.83	-4.95	> -4.00
BT-549	-7.04	-5.98	-5.32
T-47D	-6.25	-4.99	-4.24
MDA-MB-468	-6.66	-5.61	-5.11
MID	-7.23	-5.66	-4.78
Delta	0.77	2.26	0.76
Range	2.03	3.82	1.54

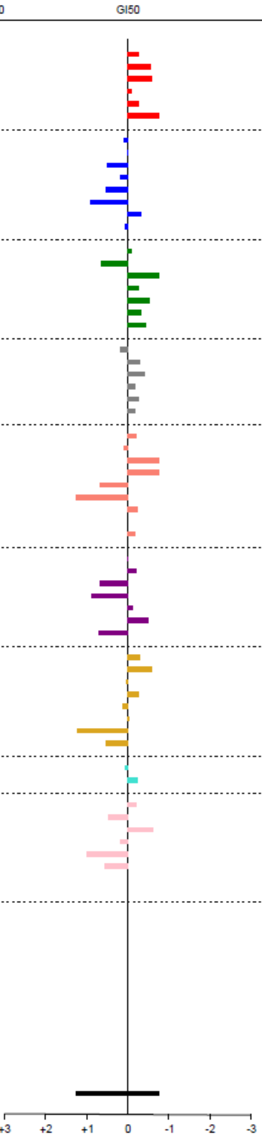

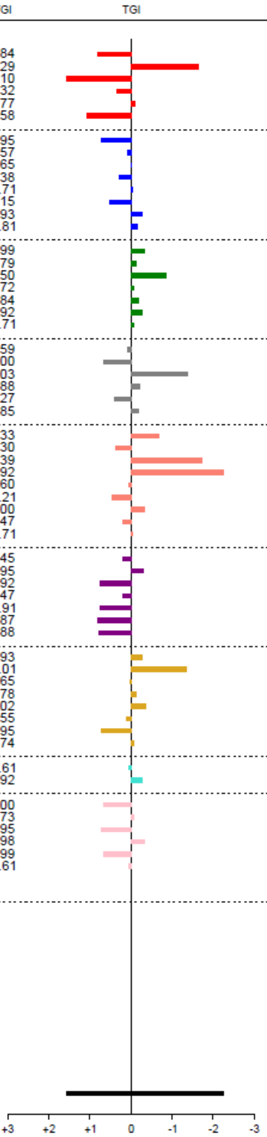

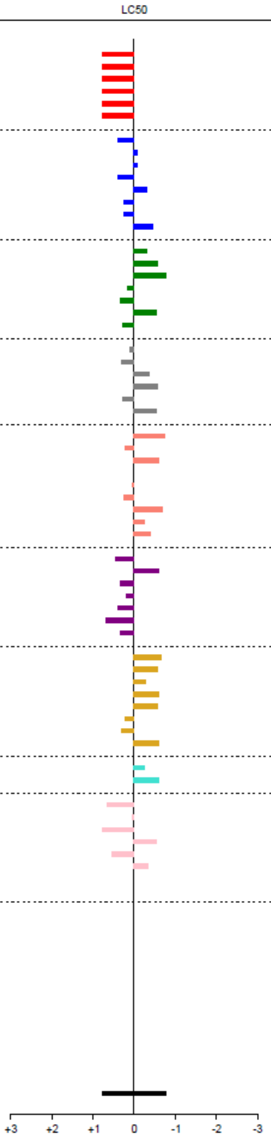

Fig. S4

Developmental Therapeutics Program One Dose Mean Graph	NSC: D-795902 / 1	Conc: 1.00E-5 Molar	Test Date: Feb 21, 2017
	Experiment ID: 1702OS60		Report Date: May 08, 2017

Panel/Cell Line	Growth Percent
**Leukemia**	
CCRF-CEM	-0.72
HL-60(TB)	-7.94
K-562	5.07
MOLT-4	5.16
RPMI-8226	-12.05
SR	4.00
**Non-Small Cell Lung Cancer**	
A549/ATCC	16.79
EKVX	-1.60
HOP-62	-33.55
HOP-92	0.54
NCI-H226	8.19
NCI-H23	-55.25
NCI-H322M	-2.24
NCI-H460	-4.96
NCI-H522	-38.59
**Colon Cancer**	
COLO 205	-50.77
HCC-2998	-97.56
HCT-116	-64.65
HCT-15	-58.85
HT29	-44.60
KM12	-45.35
SW-620	4.20
**CNS Cancer**	
SF-268	-10.76
SF-295	1.85
SF-539	-29.87
SNB-19	-72.88
SNB-75	21.03
U251	-92.22
**Melanoma**	
LOX IMVI	-81.80
MALME-3M	-39.05
M14	-20.38
MDA-MB-435	5.11
SK-MEL-2	4.96
SK-MEL-28	-3.66
SK-MEL-5	-78.33
UACC-257	-9.05
UACC-62	-65.28
**Ovarian Cancer**	
IGROV1	1.32
OVCAR-3	-30.68
OVCAR-4	32.27
OVCAR-5	5.93
OVCAR-8	7.43
NCI/ADR-RES	-4.24
SK-OV-3	18.84
**Renal Cancer**	
786-0	-21.74
A498	-14.68
ACHN	-5.01
RXF 393	-69.39
SN12C	-20.86
TK-10	15.01
UO-31	-97.70
**Prostate Cancer**	
PC-3	14.87
DU-145	-36.97
**Breast Cancer**	
MCF7	3.40
MDA-MB-231/ATCC	-52.81
HS 578T	60.08
BT-549	-19.16
T-47D	4.65
MDA-MB-468	-20.22
Mean	-19.91
Delta	77.79
Range	157.78

Mean Growth Percent - Growth Percent

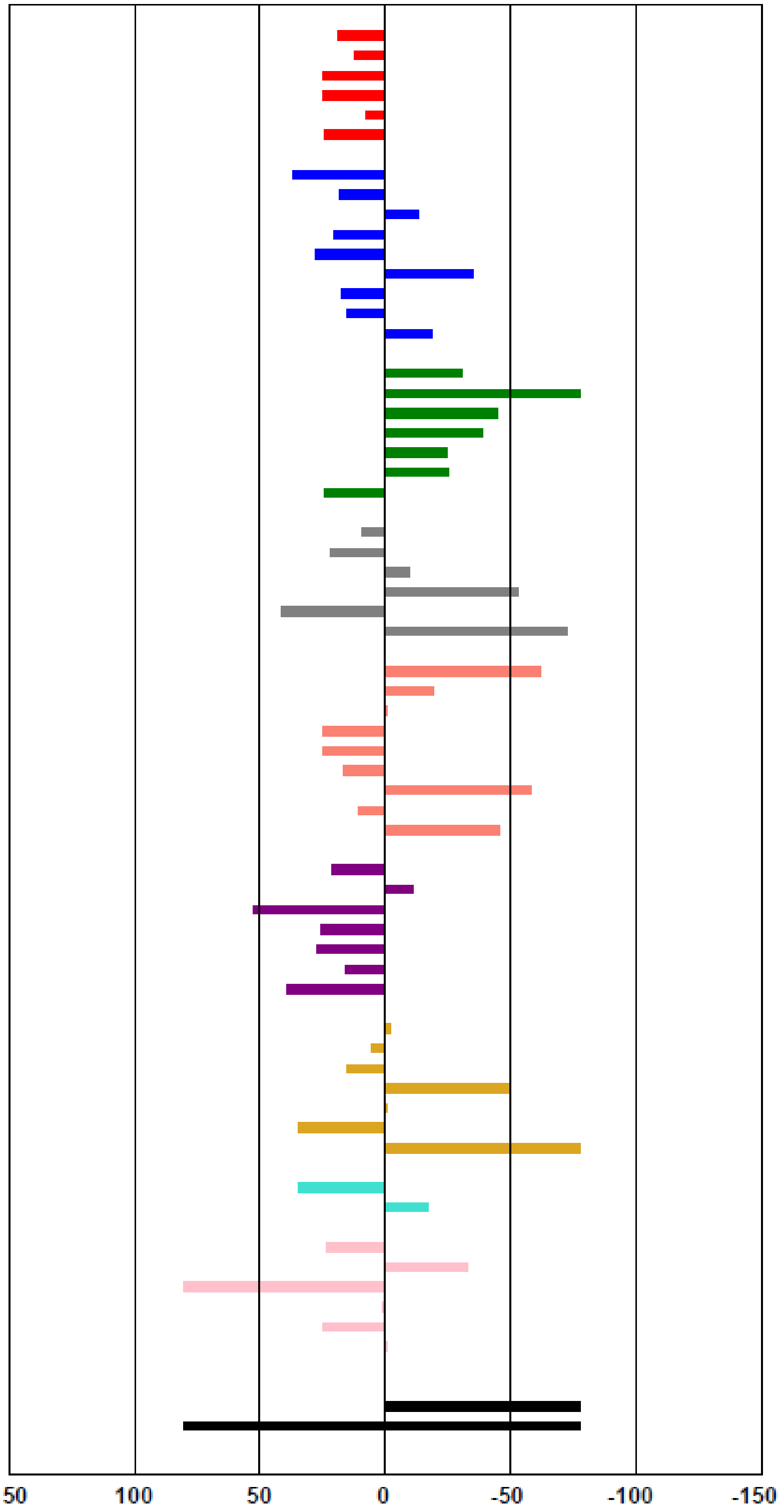

Fig. S5

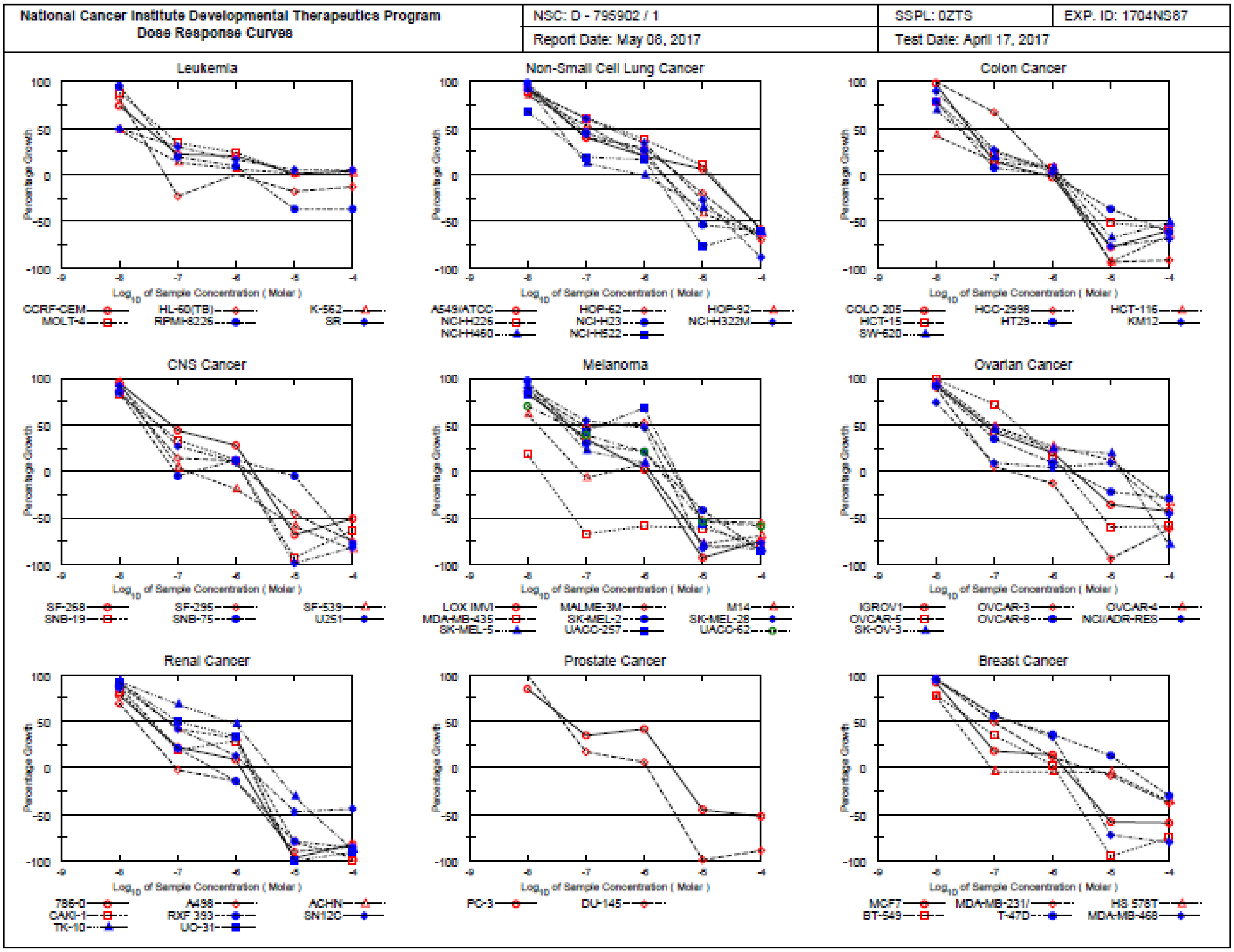
National Cancer Institute Developmental Therapeutics Program
Dose Response Curves
NSC: D - 795902 / 1
SSPL: 0ZTS
EXP. ID: 1704NS87
Report Date: May 08, 2017
Test Date: April 17, 2017
Leukemia
CCRF-CEM
HL-60(TB)
K-562
MOLT-4
RPMI-8226
SR
Non-Small Cell Lung Cancer
A549/ATCC
HOP-62
HOP-92
NCI-H226
NCI-H23
NCI-H322M
NCI-H460
NCI-H522
Colon Cancer
COLO 205
HCC-2998
HCT-116
HCT-15
HT29
KM12
SW-620
CNS Cancer
SF-268
SF-295
SF-539
SNB-19
SNB-75
U251
Melanoma
LOX IMVI
MALME-3M
M14
MDA-MB-435
SK-MEL-2
SK-MEL-28
SK-MEL-5
UACC-257
UACC-62
Ovarian Cancer
IGROV1
OVCAR-3
OVCAR-4
OVCAR-5
OVCAR-8
NCI/ADR-RES
SK-OV-3
Renal Cancer
786-0
A498
ACHN
CAKI-1
RXF 393
SN12C
TK-10
UO-31
Prostate Cancer
PC-3
DU-145
Breast Cancer
MCF7
MDA-MB-231/
HS 578T
BT-549
T-47D
MDA-MB-468
Percentage Growth
Log10 of Sample Concentration (Molar)

Fig. S6

National Cancer Institute Developmental Therapeutics Program
In-Vitro Testing Results

NSC : D - 795902 / 1	Experiment ID : 1704NS87	Test Type : 08	Units : Molar
Report Date : May 08, 2017	Test Date : April 17, 2017	QNS :	MC :
COMI : NO	Stain Reagent : SRB Dual-Pass Related	SSPL : 0ZTS	

			Log10 Concentration												
	Time		Mean Optical Densities					Percent Growth							
Panel/Cell Line	Zero	Ctrl	-8.0	-7.0	-6.0	-5.0	-4.0	-8.0	-7.0	-6.0	-5.0	-4.0	GI50	TGI	LC50
Leukemia															
CCRF-CEM	0.434	1.269	1.056	0.614	0.600	0.446	0.464	74	22	20	1	4	2.90E-8	> 1.00E-4	> 1.00E-4
HL-60(TB)	0.750	2.172	1.915	0.579	0.753	0.617	0.655	82	-23	.	-18	-13	2.02E-8	.	> 1.00E-4
K-562	0.269	1.835	1.019	0.475	0.361	0.287	0.289	48	13	6	1	1	< 1.00E-8	> 1.00E-4	> 1.00E-4
MOLT-4	0.735	2.752	2.506	1.422	1.223	0.735	0.729	88	34	24	.	.	5.05E-8	9.94E-6	> 1.00E-4
RPMI-8226	0.587	2.012	1.938	0.862	0.709	0.368	0.372	95	19	9	-37	-37	3.92E-8	1.54E-6	> 1.00E-4
SR	0.441	1.866	1.134	0.873	0.675	0.518	0.513	49	30	16	5	5	< 1.00E-8	> 1.00E-4	> 1.00E-4
Non-Small Cell Lung Cancer															
A549/ATCC	0.361	1.554	1.499	0.844	0.601	0.437	0.150	95	40	20	6	-59	6.70E-8	1.25E-5	7.37E-5
HOP-62	0.745	2.027	1.924	1.255	1.123	0.596	0.224	92	40	29	-20	-70	6.37E-8	3.93E-6	3.98E-5
HOP-92	1.114	1.590	1.517	1.364	1.204	0.648	0.402	85	53	19	-42	-64	1.19E-7	2.05E-6	2.33E-5
NCI-H226	0.841	1.849	1.746	1.455	1.227	0.946	0.340	90	61	38	10	-60	3.04E-7	1.41E-5	7.29E-5
NCI-H23	0.511	1.928	1.894	1.153	0.876	0.236	0.199	98	45	26	-54	-61	8.13E-8	2.11E-6	8.95E-6
NCI-H322M	0.874	2.134	2.029	1.629	1.299	0.636	0.095	92	60	34	-27	-89	2.39E-7	3.58E-6	2.33E-5
NCI-H460	0.221	2.208	2.076	0.450	0.218	0.141	0.085	93	12	-1	-36	-62	3.39E-8	7.85E-7	3.50E-5
NCI-H522	0.892	2.278	1.833	1.152	1.112	0.210	0.361	68	19	16	-77	-60	2.31E-8	1.49E-6	5.16E-6
Colon Cancer															
COLO 205	0.508	1.912	1.884	0.700	0.492	0.113	0.201	98	14	-3	-78	-60	3.71E-8	6.43E-7	4.23E-6
HCC-2998	0.694	2.208	2.221	1.710	0.722	0.039	0.053	101	67	2	-94	-92	1.83E-7	1.04E-6	3.45E-6
HCT-116	0.195	1.444	0.725	0.374	0.281	0.012	0.068	42	14	7	-94	-65	< 1.00E-8	1.17E-6	3.67E-6
HCT-15	0.192	1.373	1.120	0.473	0.277	0.093	0.081	79	24	7	-52	-58	3.32E-8	1.33E-6	9.41E-6
HT29	0.230	1.531	1.259	0.327	0.231	0.145	0.087	79	7	.	-37	-62	2.54E-8	1.00E-6	3.26E-5
KM12	0.508	2.654	2.430	1.096	0.552	0.115	0.160	90	27	2	-77	-69	4.33E-8	1.06E-6	4.52E-6
SW-620	0.265	1.760	1.293	0.547	0.376	0.084	0.127	69	19	7	-68	-52	2.38E-8	1.25E-6	5.73E-6
CNS Cancer															
SF-268	0.537	1.906	1.835	1.134	0.918	0.171	0.264	95	44	28	-68	-51	7.50E-8	1.95E-6	6.47E-6
SF-295	0.604	2.245	2.032	0.829	0.784	0.327	0.152	87	14	11	-46	-75	3.20E-8	1.56E-6	1.39E-5
SF-539	1.012	2.643	2.569	1.079	0.822	0.412	0.164	95	4	-19	-59	-84	3.14E-8	1.51E-7	5.88E-6
SNB-19	0.445	1.838	1.593	0.916	0.619	0.029	0.164	82	34	12	-93	-63	4.64E-8	1.31E-6	3.89E-6
SNB-75	0.886	1.765	1.633	0.844	0.990	0.839	0.196	85	-5	12	-5	-78	2.45E-8	.	4.12E-5
U251	0.334	1.582	1.477	0.676	0.460	0.005	0.060	92	27	10	-99	-82	4.44E-8	1.24E-6	3.57E-6
Melanoma															
LOX IMVI	0.333	2.340	2.023	1.033	0.378	0.025	0.083	84	35	2	-93	-75	4.93E-8	1.06E-6	3.55E-6
MALME-3M	0.572	1.299	1.194	0.923	0.948	0.267	0.253	86	48	52	-53	-56	.	3.10E-6	9.30E-6
M14	0.420	1.578	1.129	0.392	0.512	0.091	0.129	61	-7	8	-78	-69	1.46E-8	.	4.69E-6
MDA-MB-435	0.592	2.454	0.925	0.195	0.244	0.230	0.128	18	-67	-59	-61	-78	< 1.00E-8	1.62E-8	6.29E-8
SK-MEL-2	1.031	2.113	2.075	1.357	1.255	0.598	0.160	97	30	21	-42	-85	5.02E-8	2.14E-6	1.54E-5
SK-MEL-28	0.753	2.297	2.147	1.586	1.484	0.131	0.170	90	54	47	-83	-77	3.96E-7	2.31E-6	5.61E-6
SK-MEL-5	0.632	3.062	2.843	1.170	0.849	0.132	0.100	91	22	9	-79	-84	3.94E-8	1.26E-6	4.66E-6
UACC-257	1.062	1.994	1.827	1.466	1.692	0.472	0.165	82	43	68	-56	-85	.	3.54E-6	9.01E-6
UACC-62	0.631	2.492	1.928	1.358	1.017	0.290	0.260	70	39	21	-54	-59	4.39E-8	1.89E-6	8.83E-6
Ovarian Cancer															
IGROV1	0.375	1.806	1.700	0.975	0.668	0.242	0.213	93	42	20	-36	-43	6.93E-8	2.32E-6	> 1.00E-4
OVCAR-3	0.417	1.442	1.337	0.473	0.363	0.027	0.157	90	5	-13	-94	-62	2.96E-8	1.96E-7	2.87E-6
OVCAR-4	0.826	1.698	1.666	1.245	1.064	0.932	0.547	96	48	27	12	-34	9.12E-8	1.84E-5	> 1.00E-4
OVCAR-5	0.581	1.374	1.364	1.156	0.710	0.232	0.239	99	72	16	-60	-59	2.51E-7	1.63E-6	7.36E-6
OVCAR-8	0.581	2.291	2.147	1.185	0.739	0.451	0.411	92	35	9	-22	-29	5.48E-8	1.96E-6	> 1.00E-4
NCI/ADR-RES	0.487	1.646	1.346	0.589	0.534	0.587	0.265	74	9	4	9	-46	2.34E-8	1.44E-5	> 1.00E-4
SK-OV-3	0.894	1.912	1.844	1.354	1.140	1.089	0.190	93	45	24	19	-79	7.95E-8	1.57E-5	5.08E-5
Renal Cancer															
786-0	0.403	1.832	1.519	0.713	0.533	0.011	0.073	78	22	9	-97	-82	3.15E-8	1.22E-6	3.59E-6
A498	1.422	2.143	1.923	1.400	1.227	0.137	0.207	69	-2	-14	-90	-85	1.88E-8	9.50E-8	2.97E-6
ACHN	0.379	1.661	1.554	0.914	0.794	0.075	0.012	92	42	32	-80	-97	6.83E-8	1.94E-6	5.38E-6
CAKI-1	0.767	2.742	2.381	1.143	1.336	-0.005	-0.010	82	19	29	-100	-100	3.21E-8	1.67E-6	4.09E-6
RXF 393	0.685	1.483	1.378	0.852	0.593	0.147	0.099	87	21	-14	-79	-86	3.62E-8	4.05E-7	3.64E-6
SN12C	0.391	1.745	1.591	0.962	0.574	0.206	0.219	89	42	13	-47	-44	6.77E-8	1.66E-6	> 1.00E-4
TK-10	0.865	1.748	1.682	1.466	1.279	0.598	0.104	93	68	47	-31	-88	7.11E-7	4.01E-6	2.16E-5
UO-31	0.608	2.186	2.065	1.395	1.137	-0.004	0.053	92	50	34	-100	-91	9.94E-8	1.78E-6	4.22E-6
Prostate Cancer															
PC-3	0.440	1.465	1.307	0.794	0.866	0.244	0.210	85	35	42	-45	-52	4.90E-8	3.03E-6	5.03E-5
DU-145	0.338	1.512	1.515	0.542	0.411	0.003	0.036	100	17	6	-99	-89	4.04E-8	1.15E-6	3.41E-6
Breast Cancer															
MCF7	0.346	2.134	1.997	0.673	0.597	0.147	0.142	92	18	14	-58	-59	3.73E-8	1.57E-6	7.82E-6
MDA-MB-231/ATCC	0.547	1.485	1.450	1.006	0.653	0.502	0.337	96	49	11	-8	-38	9.50E-8	3.76E-6	> 1.00E-4
HS 578T	0.815	1.560	1.387	0.783	0.779	0.774	0.524	77	-4	-4	-5	-36	2.14E-8	8.93E-8	> 1.00E-4
BT-549	1.139	1.958	1.777	1.426	1.165	0.061	0.281	78	35	3	-95	-75	4.47E-8	1.08E-6	3.50E-6
T-47D	0.703	1.601	1.555	1.204	1.024	0.819	0.496	95	56	36	13	-30	1.94E-7	2.01E-5	> 1.00E-4
MDA-MB-468	0.640	1.400	1.366	1.071	0.888	0.182	0.130	96	57	33	-72	-80	1.89E-7	2.05E-6	6.20E-6

Fig. S7

National Cancer Institute Developmental Therapeutics Program<br>Mean Graphs	NSC : D - 795902/1	Units :Molar	SSPL :0ZTS	EXP. ID :1704NS87
	Report Date :May 08, 2017		Test Date :April 17, 2017	

Panel/Cell Line	Log_{10}GI50	Log_{10}TGI	Log_{10}LC50
**Leukemia**			
CCRF-CEM	-7.54	> -4.00	> -4.00
HL-60(TB)	-7.70		> -4.00
K-562	< -8.00	> -4.00	> -4.00
MOLT-4	-7.30	-5.00	> -4.00
RPMI-8226	-7.41	-5.81	> -4.00
SR	< -8.00	> -4.00	> -4.00
**Non-Small Cell Lung Cancer**			
A549/ATCC	-7.17	-4.90	-4.13
HOP-62	-7.20	-5.41	-4.40
HOP-92	-6.92	-5.69	-4.63
NCI-H226	-6.52	-4.85	-4.14
NCI-H23	-7.09	-5.68	-5.05
NCI-H322M	-6.62	-5.45	-4.63
NCI-H460	-7.47	-6.11	-4.46
NCI-H522	-7.64	-5.83	-5.29
**Colon Cancer**			
COLO 205	-7.43	-6.19	-5.37
HCC-2998	-6.74	-5.98	-5.46
HCT-116	< -8.00	-5.93	-5.44
HCT-15	-7.48	-5.88	-5.03
HT29	-7.59	-6.00	-4.49
KM12	-7.36	-5.97	-5.34
SW-620	-7.62	-5.90	-5.24
**CNS Cancer**			
SF-268	-7.13	-5.71	-5.19
SF-295	-7.50	-5.81	-4.86
SF-539	-7.50	-6.82	-5.23
SNB-19	-7.33	-5.88	-5.41
SNB-75	-7.61		-4.38
U251	-7.35	-5.91	-5.45
**Melanoma**			
LOX IMVI	-7.31	-5.98	-5.45
MALME-3M		-5.51	-5.03
M14	-7.84		-5.33
MDA-MB-435	< -8.00	-7.79	-7.20
SK-MEL-2	-7.30	-5.67	-4.81
SK-MEL-28	-6.40	-5.64	-5.25
SK-MEL-5	-7.40	-5.90	-5.33
UACC-257		-5.45	-5.06
UACC-62	-7.36	-5.72	-5.05
**Ovarian Cancer**			
IGROV1	-7.16	-5.64	> -4.00
OVCAR-3	-7.53	-6.71	-5.54
OVCAR-4	-7.04	-4.74	> -4.00
OVCAR-5	-6.60	-5.79	-5.13
OVCAR-8	-7.26	-5.71	> -4.00
NCI/ADR-RES	-7.63	-4.84	> -4.00
SK-OV-3	-7.10	-4.80	-4.29
**Renal Cancer**			
786-0	-7.50	-5.91	-5.45
A498	-7.73	-7.02	-5.53
ACHN	-7.17	-5.71	-5.27
CAKI-1	-7.49	-5.78	-5.39
RXF 393	-7.44	-6.39	-5.44
SN12C	-7.17	-5.78	> -4.00
TK-10	-6.15	-5.40	-4.66
UO-31	-7.00	-5.75	-5.37
**Prostate Cancer**			
PC-3	-7.31	-5.52	-4.30
DU-145	-7.39	-5.94	-5.47
**Breast Cancer**			
MCF7	-7.43	-5.80	-5.11
MDA-MB-231/ATCC	-7.02	-5.43	> -4.00
HS 578T	-7.67	-7.06	> -4.00
BT-549	-7.35	-5.97	-5.46
T-47D	-6.71	-4.70	> -4.00
MDA-MB-468	-6.72	-5.69	-5.21
_MID	-7.31	-5.69	-4.84
Delta	0.69	2.1	2.36
Range	1.86	3.79	3.2

GI50

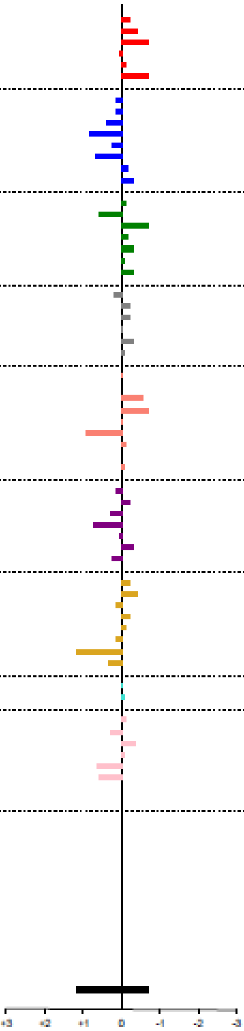

TGI

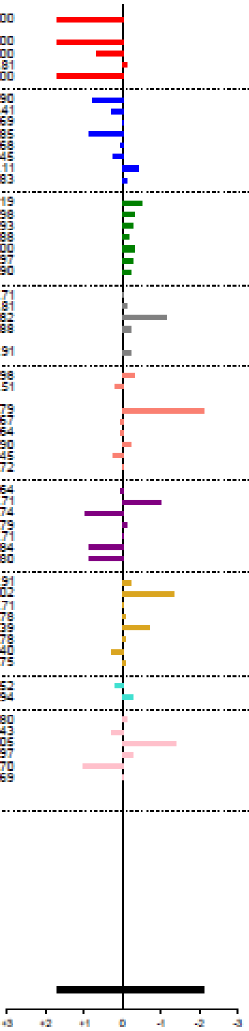

LC50

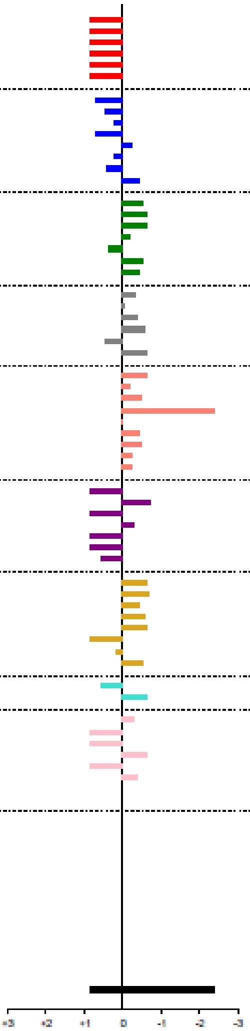

Fig. S8

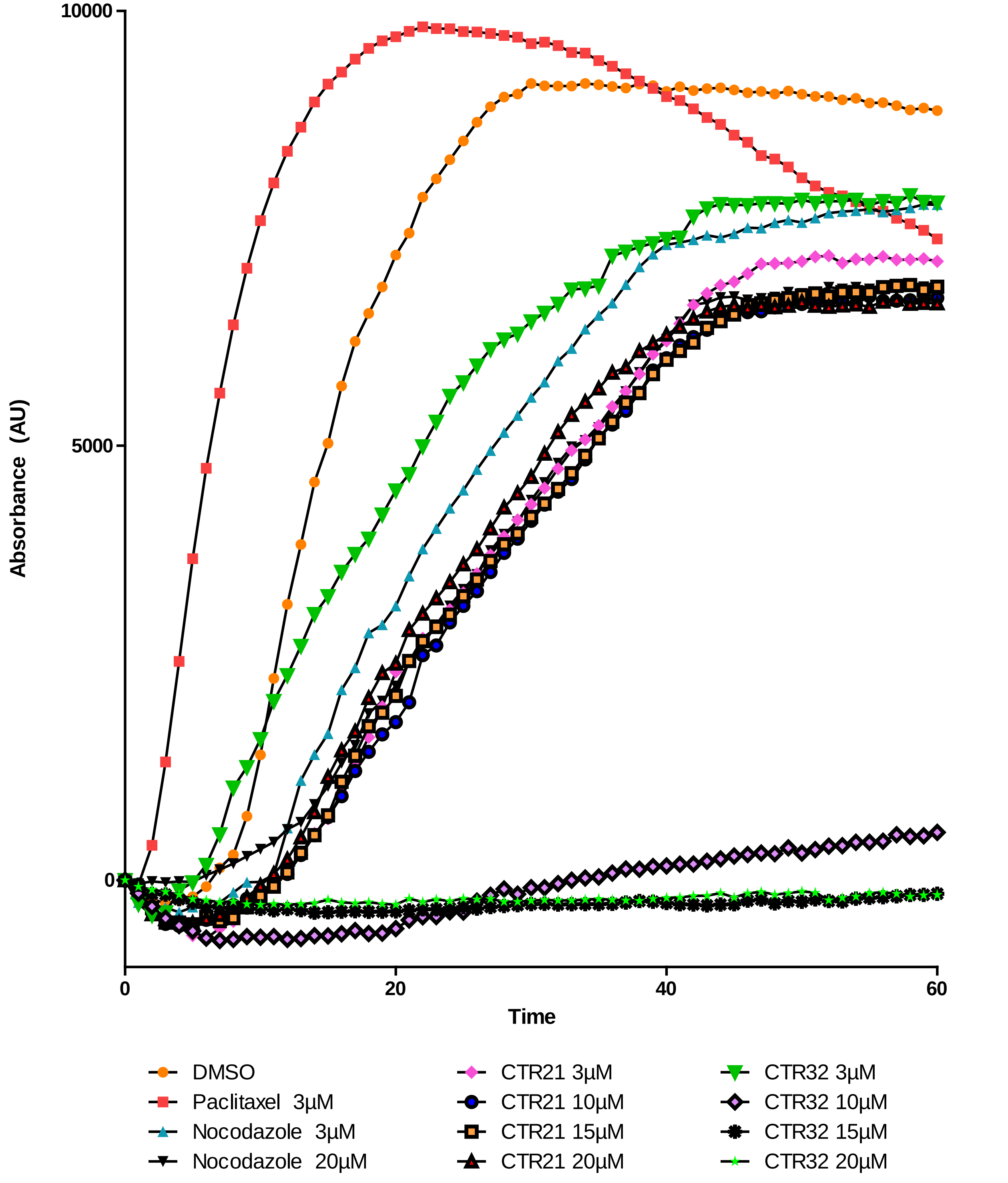
10000
5000
0
Absorbance (AU)
0
20
40
60
Time
DMSO
Paclitaxel 3µM
Nocodazole 3µM
Nocodazole 20µM
CTR21 3µM
CTR21 10µM
CTR21 15µM
CTR21 20µM
CTR32 3µM
CTR32 10µM
CTR32 15µM
CTR32 20µM

Fig. S9.

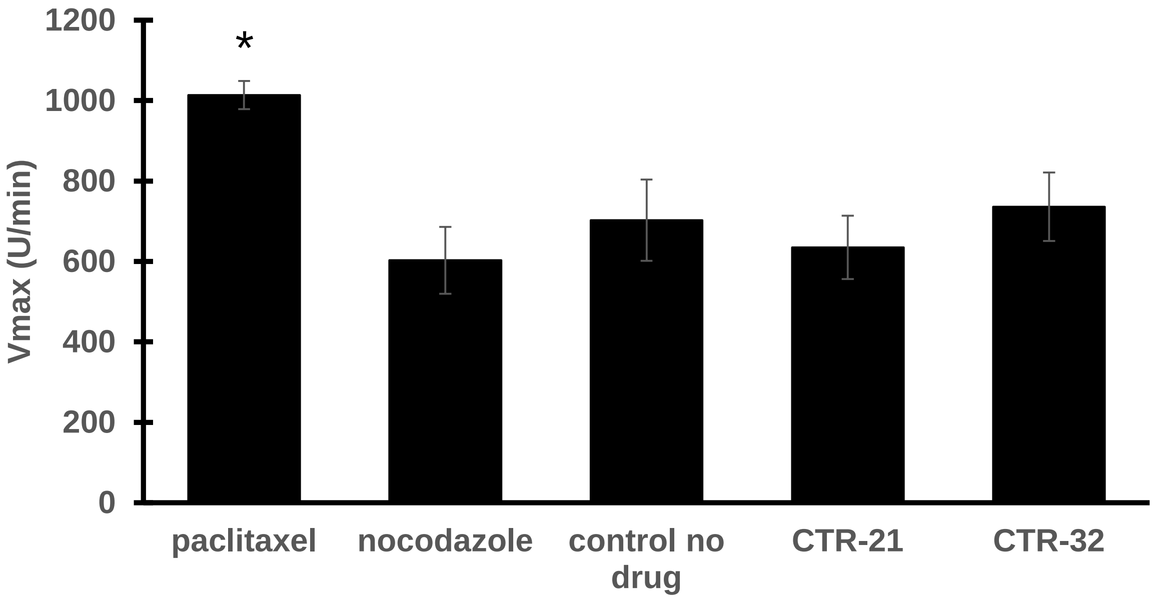
1200
1000
800
600
400
200
0
Vmax (U/min)
*
paclitaxel
nocodazole
control no drug
CTR-21
CTR-32

Fig. S10

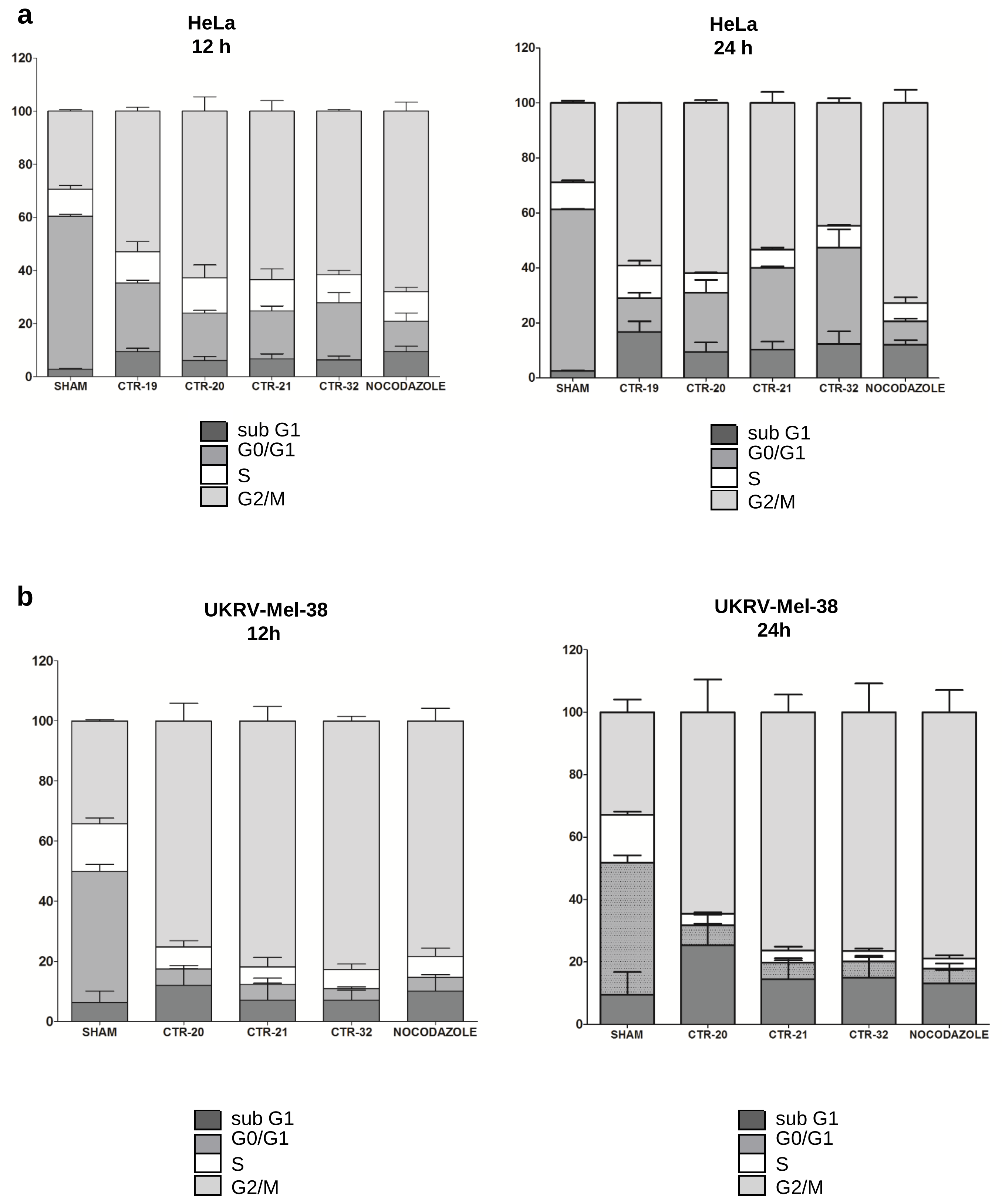

a
HeLa
12 h
120
100
80
60
40
20
0
SHAM
CTR-19
CTR-20
CTR-21
CTR-32
NOCODAZOLE
sub G1
G0/G1
S
G2/M
HeLa
24 h
b
UKRV-Mel-38
12h
UKRV-Mel-38
24h

Fig. S11

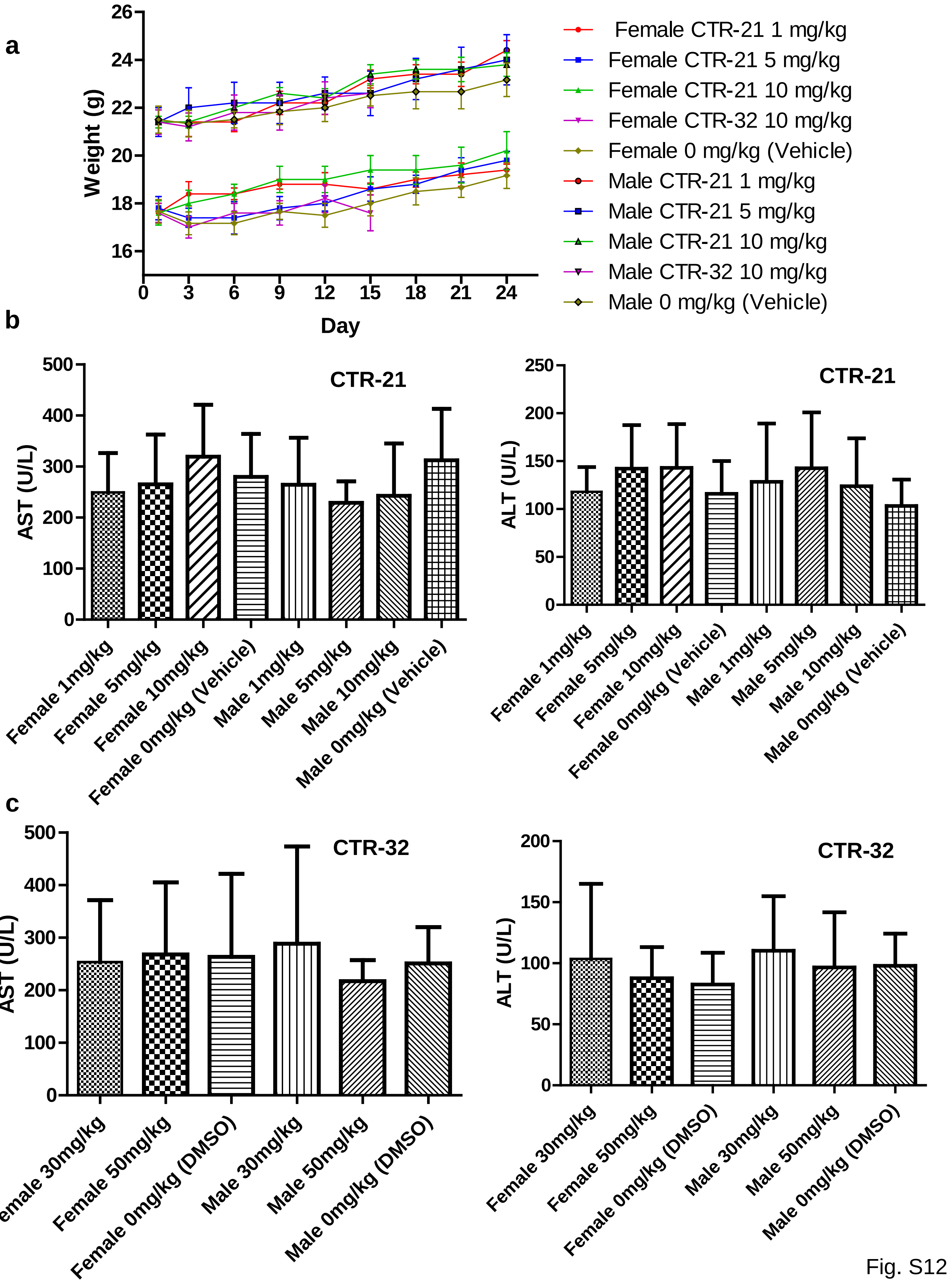
a
Weight (g)
Day
Female CTR-21 1 mg/kg
Female CTR-21 5 mg/kg
Female CTR-21 10 mg/kg
Female CTR-32 10 mg/kg
Female 0 mg/kg (Vehicle)
Male CTR-21 1 mg/kg
Male CTR-21 5 mg/kg
Male CTR-21 10 mg/kg
Male CTR-32 10 mg/kg
Male 0 mg/kg (Vehicle)
b
CTR-21
AST (U/L)
ALT (U/L)
Female 1mg/kg
Female 5mg/kg
Female 10mg/kg
Female 0mg/kg (Vehicle)
Male 1mg/kg
Male 5mg/kg
Male 10mg/kg
Male 0mg/kg (Vehicle)
c
CTR-32
Female 30mg/kg
Female 50mg/kg
Female 0mg/kg (DMSO)
Male 30mg/kg
Male 50mg/kg
Male 0mg/kg (DMSO)

Fig. S12
