# Supplementary figures and images for: Lead optimization of novel quinolone chalcone compounds by a structure–activity relationship (SAR) study to increase efficacy and metabolic stability
Source: Sci Rep. 2021 Nov 3;11:21576. doi: 10.1038/s41598-021-01058-z (PMC8566451; doi:10.1038/s41598-021-01058-z)

### Cleaved PARP

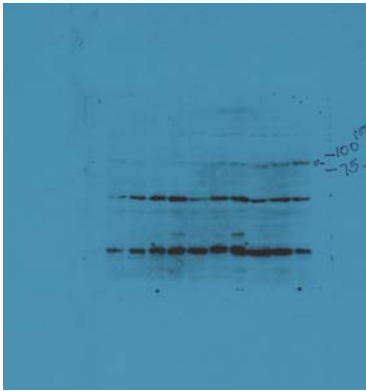

### BCL-XL

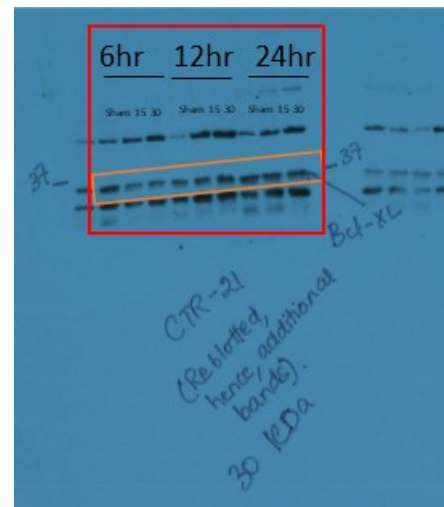

### P-BCL-XL<sup>S63</sup>

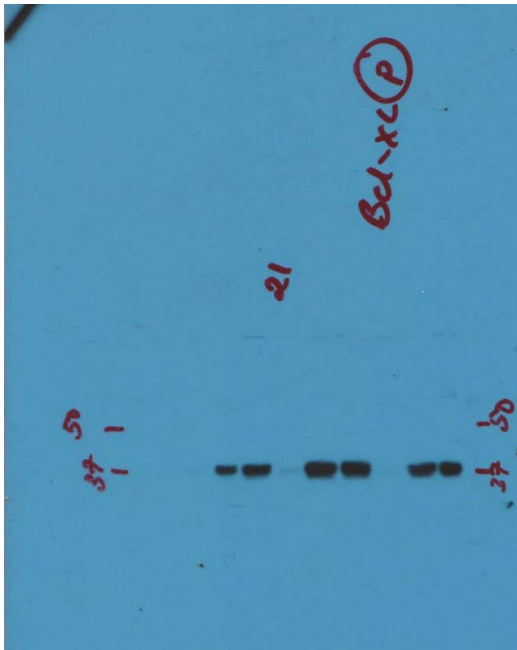

### Cyclin B1

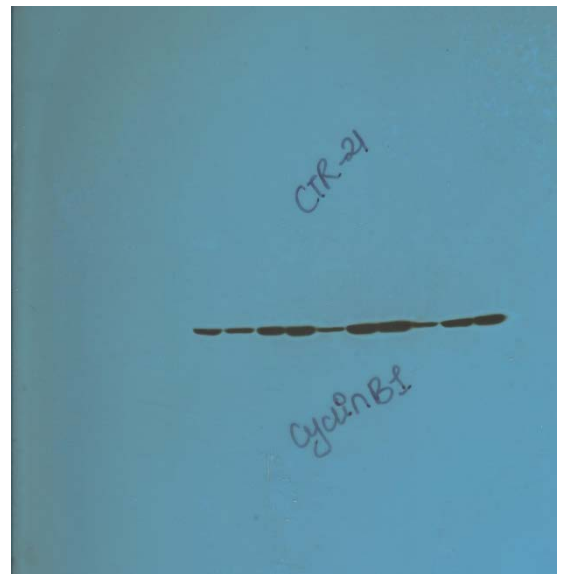

### GAPDH

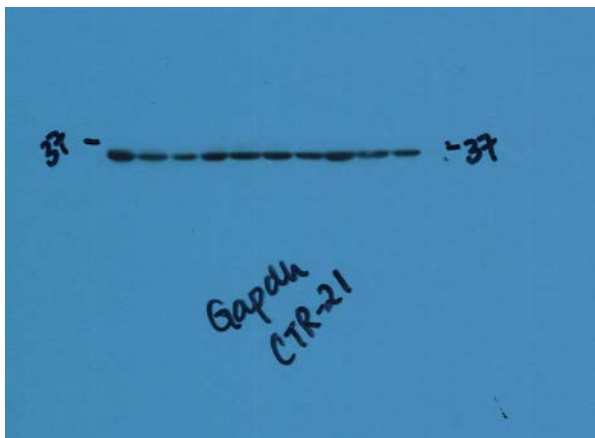

### Cdc25C

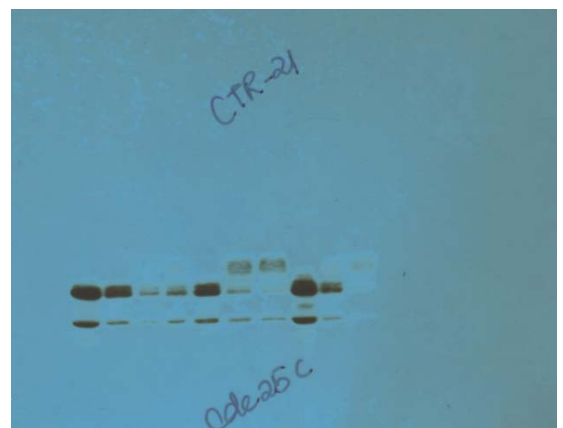

Supplement: Supplementary file 2 — Supplementary Information 2. [file 41598_2021_1058_MOESM2_ESM.pdf]
