# Supplementary material for: Lead optimization of novel quinolone chalcone compounds by a structure–activity relationship (SAR) study to increase efficacy and metabolic stability
Source: Sci Rep. 2021 Nov 3;11:21576. doi: 10.1038/s41598-021-01058-z (PMC8566451; doi:10.1038/s41598-021-01058-z)

NMR SPECTRA OF CTR-17

23-11-2020 23:36:14

Formula	$C_{19}H_{15}NO_3$	FW	305.3273

Acquisition Time (sec)	3.9977	Comment	PROTONRO DMSO /opt/topspin/nmrsu/EXTERNAL/APR16 nmrsu	Date	01 Apr 2016 15:11:04
Date Stamp	01 Apr 2016 15:11:04				
File Name	E:\DELL COMPUTER DATA\MASS DATA\NMR DATA\NEW NMR\REST OF NMR\New folder\RGPV\RGPV\RGPV-CKT-01\1\fid				
Frequency (MHz)	400.13	Nucleus	1H	Number of Transients	16	Origin	spect
Original Points Count	32768	Owner	nmrsu	Points Count	32768	Pulse Sequence	zg30
Receiver Gain	161.00	SW(cyclical) (Hz)	8196.72	Solvent	DMSO-d6	Spectrum Offset (Hz)	2384.6575
Spectrum Type	STANDARD	Sweep Width (Hz)	8196.47	Temperature (degree C)	25.060		

^{1}H NMR (400 MHz, DMSO-d_6) δ 12.01 (s, 1H), 8.42 (s, 1H), 7.82 (d, J = 16.01 Hz, 1H), 7.68 (d, J = 7.75 Hz, 1H), 7.47 - 7.57 (m, 3H), 7.43 (dd, J = 1.75, 7.50 Hz, 1H), 7.30 (d, J = 8.00 Hz, 1H), 7.12 - 7.23 (m, 2H), 7.04 (t, J = 7.50 Hz, 1H), 3.82 (s, 3H)

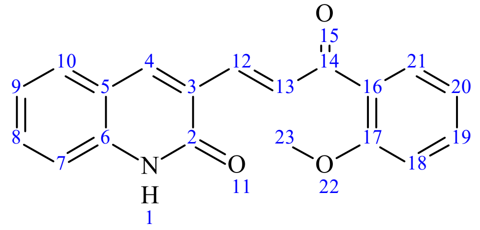

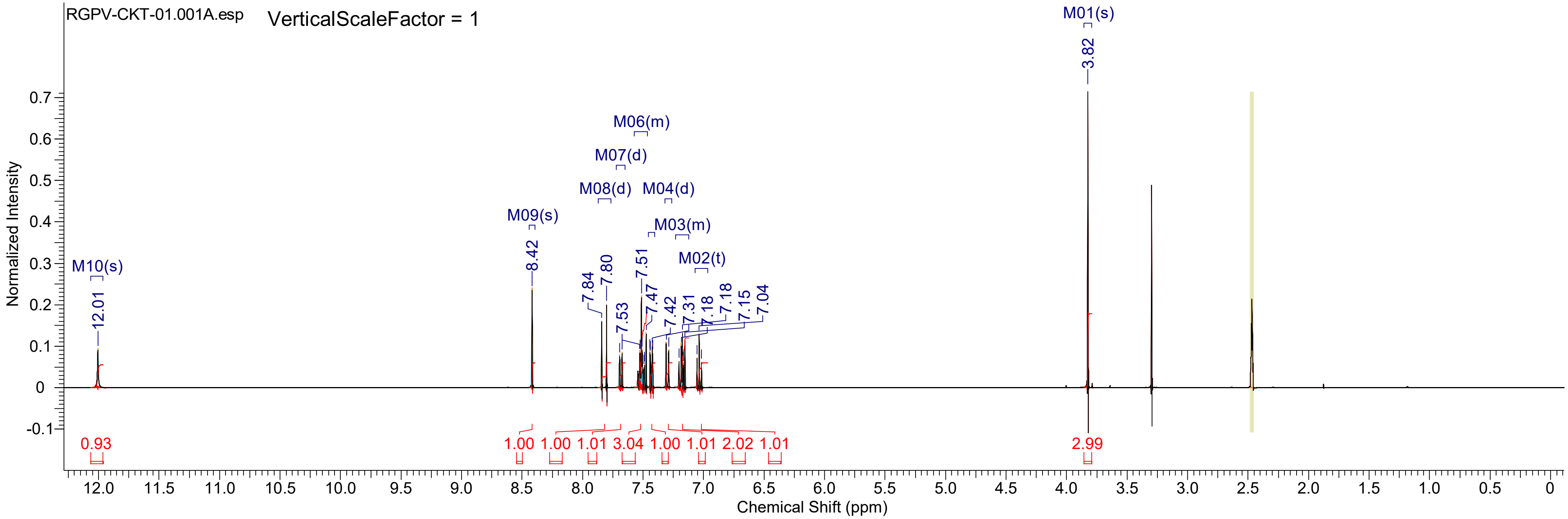

NMR SPECTRA OF CTR-18

24-11-2020 00:54:48

Formula $C_{20}H_{17}NO_3$	*FW* 319.3539

Acquisition Time (sec)	2.7263	*Comment*	CK-19	*Date*	06 Jul 2011 20:12:16				
Date Stamp	06 Jul 2011 20:12:16			*File Name*	E:\DELL COMPUTER DATA\MASS DATA\NMR DATA\CK SERIES\190\fid				
Frequency (MHz)	400.13	*Nucleus*	1H	*Number of Transients*	8	*Origin*	spect	*Original Points Count*	32768
Owner	Administrator	*Points Count*	32768	*Pulse Sequence*	zg30	*Receiver Gain*	512.00	*SW(cyclical) (Hz)*	12019.23
Solvent	DMSO-d6	*Spectrum Offset (Hz)*	2488.4370	*Spectrum Type*	STANDARD	*Sweep Width (Hz)*	12018.86	*Temperature (degree C)*	24.100

^{1}H NMR (400 MHz, DMSO-d_6) δ 11.88 (s, 1H), 8.15 (s, 1H), 7.89 (d, *J* = 15.77 Hz, 1H), 7.58 (d, *J* = 15.77 Hz, 1H), 7.47 - 7.54 (m, 2H), 7.44 (s, 1H), 7.32 (d, *J* = 8.44 Hz, 1H), 7.25 (d, *J* = 8.44 Hz, 1H), 7.10 (d, *J* = 8.44 Hz, 1H), 7.01 - 7.07 (m, 1H), 3.90 (s, 3H), 2.39 (s, 3H)

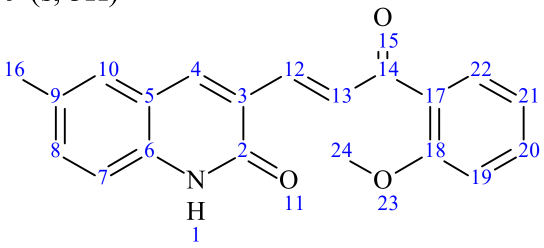

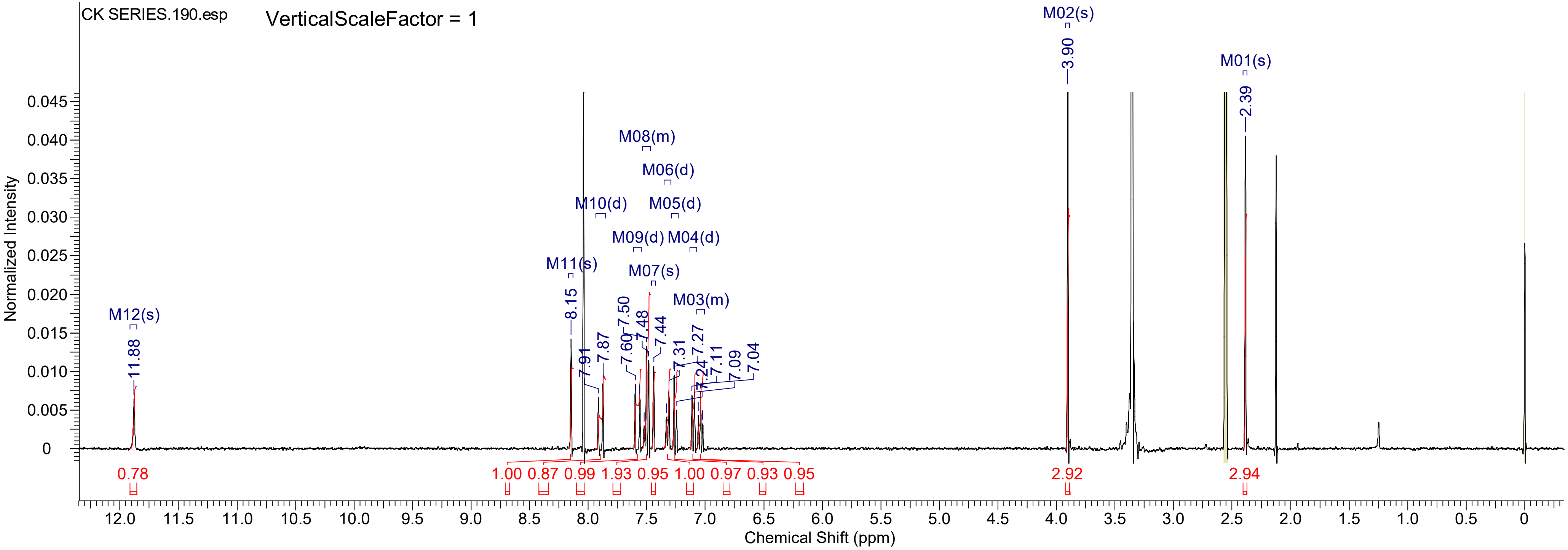

NMR SPECTRA OF CTR-19

24-11-2020 00:26:26

Formula $C_{20}H_{17}NO_4$	FW 335.3533

Acquisition Time (sec)	3.9846	Comment	EXTERNAL-CK-21			Date	09 Oct 2012 14:36:56
Date Stamp	09 Oct 2012 14:36:56			File Name	D:\AMIT PROJECTS\IND-2 GRANT\NMR DATA\CK SERIES\CK-21\EXTERNAL-CK-21\1\fid		
Frequency (MHz)	400.13	Nucleus	1H	Number of Transients	16	Origin	spect
Original Points Count	32768	Owner	nmrsu	Points Count	32768	Pulse Sequence	zg30
Receiver Gain	203.00	SW(cyclical) (Hz)	8223.68	Solvent	DMSO-d6	Spectrum Offset (Hz)	2470.9683
Spectrum Type	STANDARD	Sweep Width (Hz)	8223.43	Temperature (degree C)	25.160		

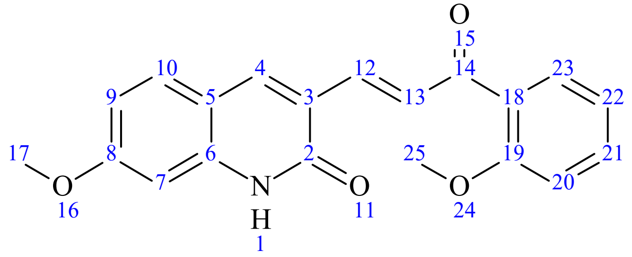

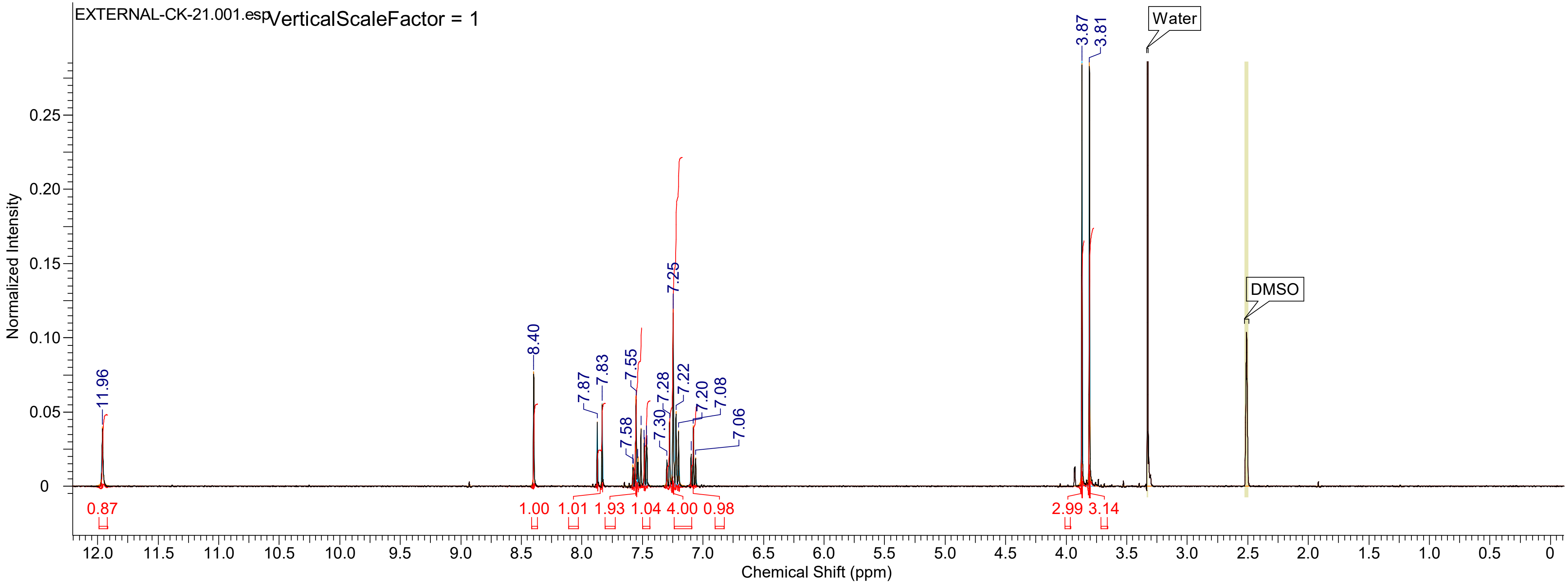

NMR SPECTRA OF CTR-21

23-11-2020 17:39:51

Formula	$C_{20}H_{17}NO_4$	*FW*	335.3533

Acquisition Time (sec)	3.9977	*Comment*	PROTONRO DMSO /opt/topspin/nmrsu/EXTERNAL/APR16 nmrsu			*Date*	01 Apr 2016 14:58:16
Date Stamp	01 Apr 2016 14:58:16						
File Name	E:\DELL COMPUTER DATA\MASS DATA\NMR DATA\NEW NMR\REST OF NMR\New folder\RGPV (1)\RGPV\RGPV-CKT-05\1\fid						
Frequency (MHz)	400.13	*Nucleus*	1H	*Number of Transients*	16	*Origin*	spect
Original Points Count	32768	*Owner*	nmrsu	*Points Count*	32768	*Pulse Sequence*	zg30
Receiver Gain	203.00	*SW(cyclical) (Hz)*	8196.72	*Solvent*	DMSO-d6	*Spectrum Offset (Hz)*	2384.6575
Spectrum Type	STANDARD	*Sweep Width (Hz)*	8196.47	*Temperature (degree C)*	25.260		

^{1}H NMR (400 MHz, DMSO-d_6) δ 11.12 (s, 1H), 8.41 (s, 1H), 7.84 (d, J = 16.01 Hz, 1H), 7.47 - 7.57 (m, 2H), 7.44 (d, J = 7.50 Hz, 1H), 7.27 (d, J = 7.25 Hz, 1H), 7.09 - 7.20 (m, 3H), 7.04 (t, J = 7.50 Hz, 1H), 3.88 (s, 3H), 3.83 (s, 3H)

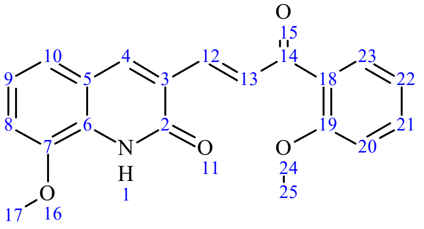

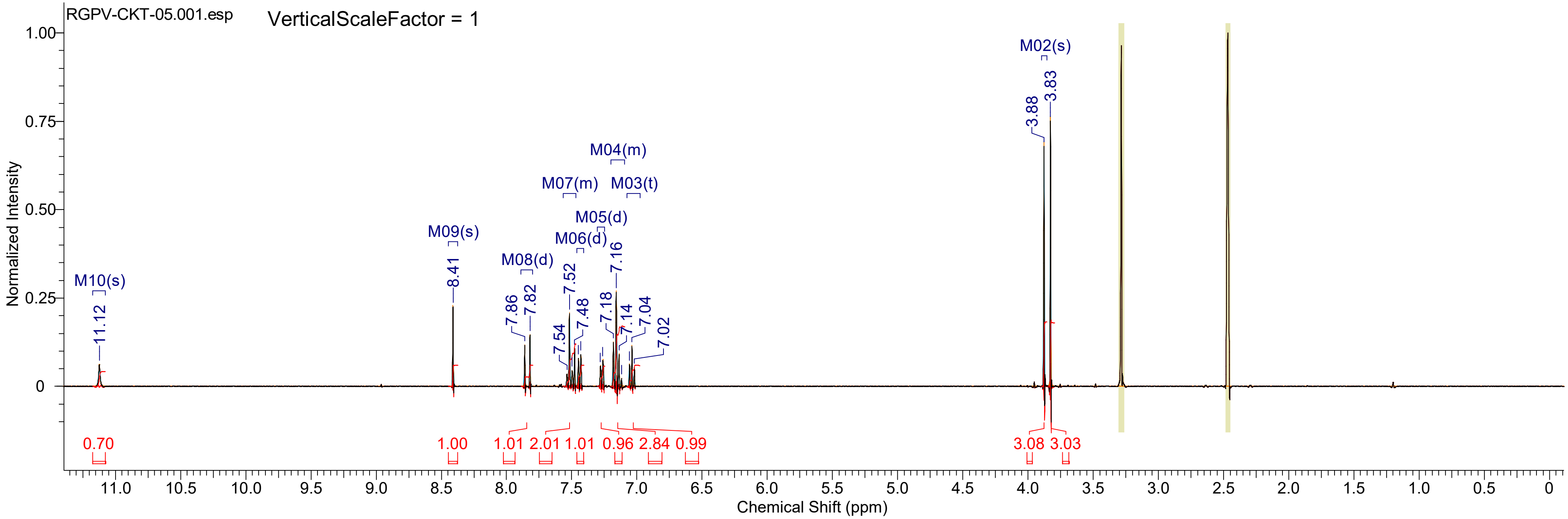

NMR SPECTRA OF CTR-25

23-11-2020 21:10:10

Formula $C_{20}H_{17}NO_4$	*FW* 335.3533

Acquisition Time (sec)	3.9977	*Comment*	PROTONRO DMSO /opt/topspin/nmrsu/EXTERNAL/DEC13 nmrsu			*Date*	21 Dec 2013 15:40:56
Date Stamp	21 Dec 2013 15:40:56						
File Name	E:\DELL COMPUTER DATA\MASS DATA\NMR DATA\NEW NMR\EXTERNAL ABL NEW\CKA-24\EXTERNAL-CKA-24\1\fid						
Frequency (MHz)	400.13	*Nucleus*	1H	*Number of Transients*	16	*Origin*	spect
Original Points Count	32768	*Owner*	nmrsu	*Points Count*	32768	*Pulse Sequence*	zg30
Receiver Gain	203.00	*SW(cyclical) (Hz)*	8196.72	*Solvent*	DMSO-d6	*Spectrum Offset (Hz)*	2384.6575
Spectrum Type	STANDARD	*Sweep Width (Hz)*	8196.47	*Temperature (degree C)*	26.860		

^{1}H NMR (400 MHz, DMSO-d_6) δ 12.00 (br. s., 1H), 8.41 (s, 1H), 7.66 (d, *J* = 8.00 Hz, 1H), 7.47 - 7.56 (m, 1H), 7.32 - 7.41 (m, 2H), 7.13 - 7.31 (m, 3H), 6.73 (d, *J* = 8.50 Hz, 2H), 3.69 (s, 6H)

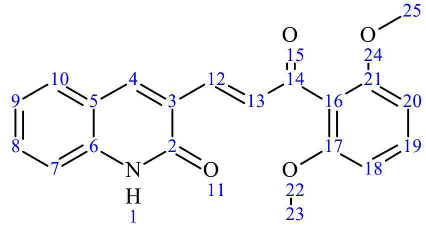

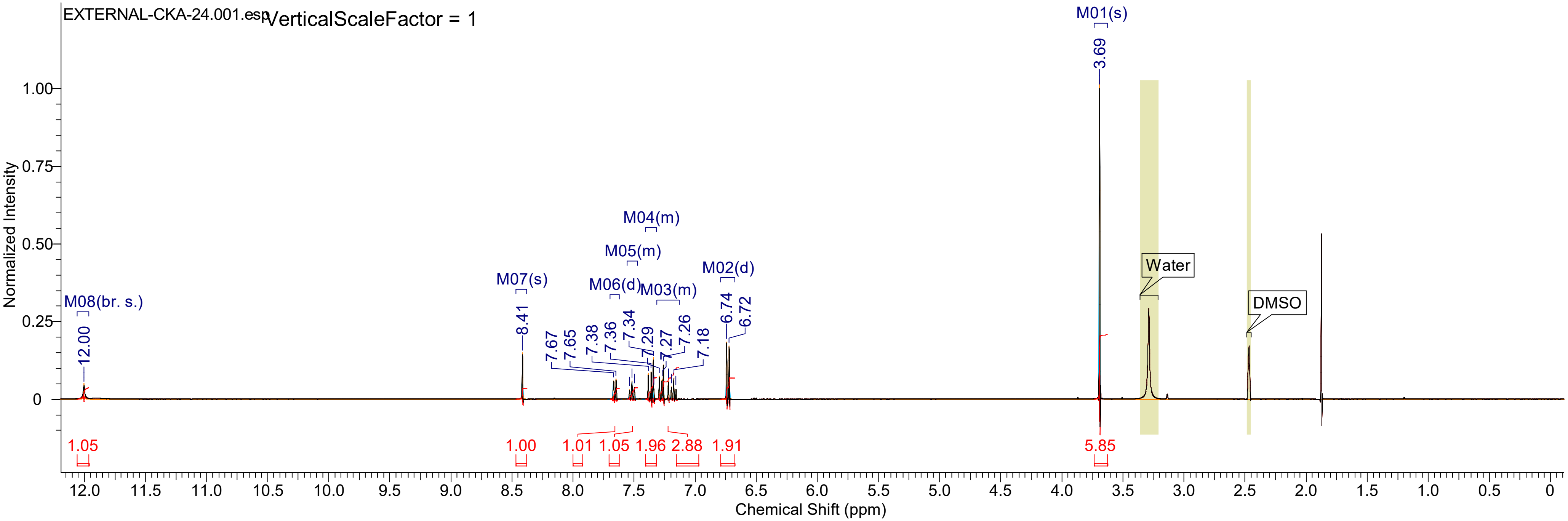

NMR SPECTRA OF CTR-32

23-11-2020 20:33:26

Formula	$C_{20}H_{17}NO_3$	FW	319.3539

Acquisition Time (sec)	3.9977	Comment	PROTONRO DMSO /opt/topspin/nmrsu/EXTERNAL/DEC13 nmrsu	Date	21 Dec 2013 15:47:20
Date Stamp	21 Dec 2013 15:47:20				
File Name	E:\DELL COMPUTER DATA\MASS DATA\NMR DATA\NEW NMR\EXTERNAL ABL NEW\CKA-22\EXTERNAL-CKA-22\1\fid				
Frequency (MHz)	400.13	Nucleus	1H	Number of Transients	16	Origin	spect
Original Points Count	32768	Owner	nmrsu	Points Count	32768	Pulse Sequence	zg30
Receiver Gain	161.00	SW(cyclical) (Hz)	8196.72	Solvent	DMSO-d6	Spectrum Offset (Hz)	2384.6575
Spectrum Type	STANDARD	Sweep Width (Hz)	8196.47	Temperature (degree C)	26.260		

^{1}H NMR (400 MHz, DMSO-d_6) δ 12.02 (s, 1H), 8.39 (s, 1H), 8.00 (d, J = 15.76 Hz, 1H), 7.68 (d, J = 8.00 Hz, 1H), 7.45 - 7.55 (m, 4H), 7.29 (d, J = 8.25 Hz, 1H), 7.12 - 7.22 (m, 2H), 7.02 (t, J = 7.38 Hz, 1H), 4.12 (d, J = 7.00 Hz, 2H), 1.31 (t, J = 6.88 Hz, 3H)

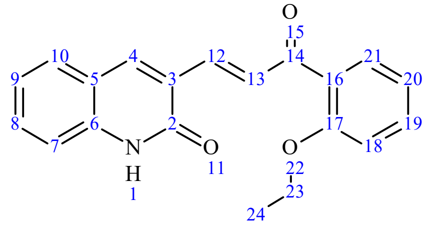

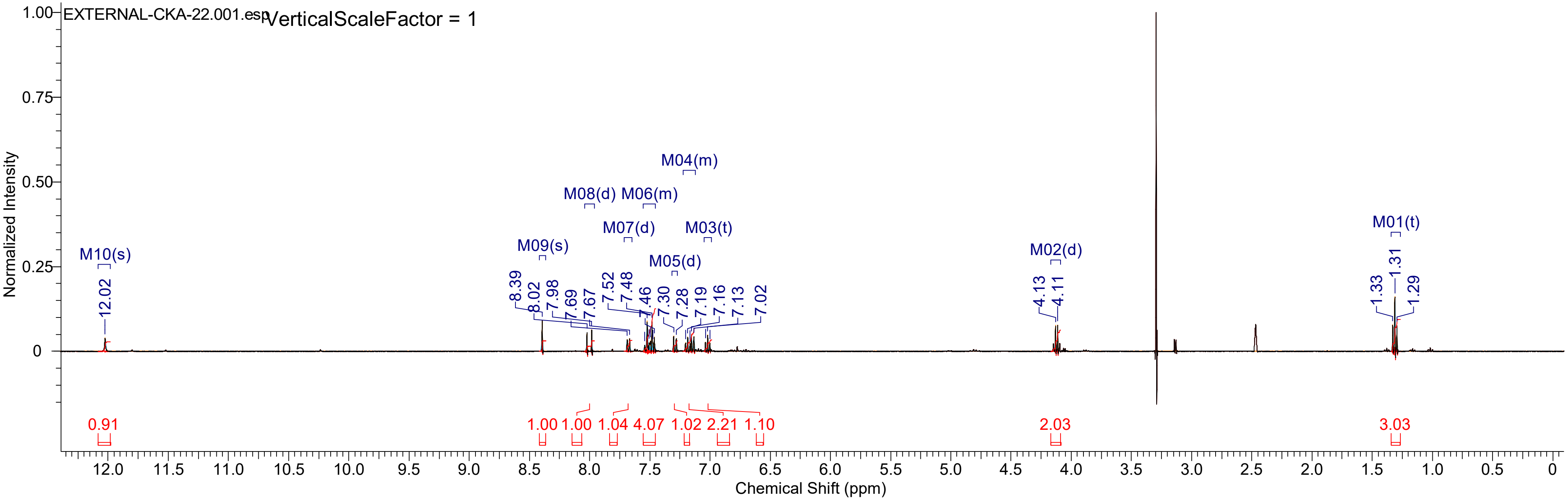

NMR SPECTRA OF CTR-33

24-11-2020 01:29:10

Formula $C_{21}H_{19}NO_5$	*FW* 365.3793

Acquisition Time (sec)	3.9977	*Comment*	PROTONRO DMSO /opt/topspin/nmrsu/EXTERNAL/MAR16 nmrsu			*Date*	14 Mar 2016 14:04:56
Date Stamp	14 Mar 2016 14:04:56						
File Name	D:\AMIT PROJECTS\IND-2 GRANT\NMR DATA\NEW NMR\NMR CKT_FEB2016\NMR fids-Spectra\NMR fids-Spectra\EX-RGPV-CKT-17\1\fid						
Frequency (MHz)	400.13	*Nucleus*	1H	*Number of Transients*	16	*Origin*	spect
Original Points Count	32768	*Owner*	nmrsu	*Points Count*	32768	*Pulse Sequence*	zg30
Receiver Gain	203.00	*SW(cyclical) (Hz)*	8196.72	*Solvent*	DMSO-d6	*Spectrum Offset (Hz)*	2384.6575
Spectrum Type	STANDARD	*Sweep Width (Hz)*	8196.47	*Temperature (degree C)*	25.060		

^{1}H NMR (400 MHz, DMSO-d_6) δ 11.92 (s, 1H), 8.35 (s, 1H), 7.35 - 7.39 (m, 1H), 7.32 - 7.35 (m, 1H), 7.16 - 7.27 (m, 4H), 6.73 (d, J = 8.50 Hz, 2H), 3.75 (s, 3H), 3.69 (s, 6H)

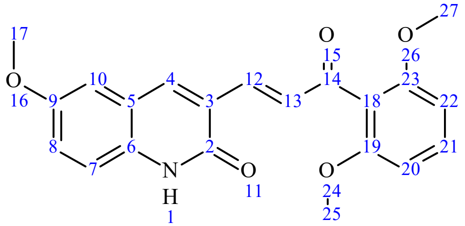

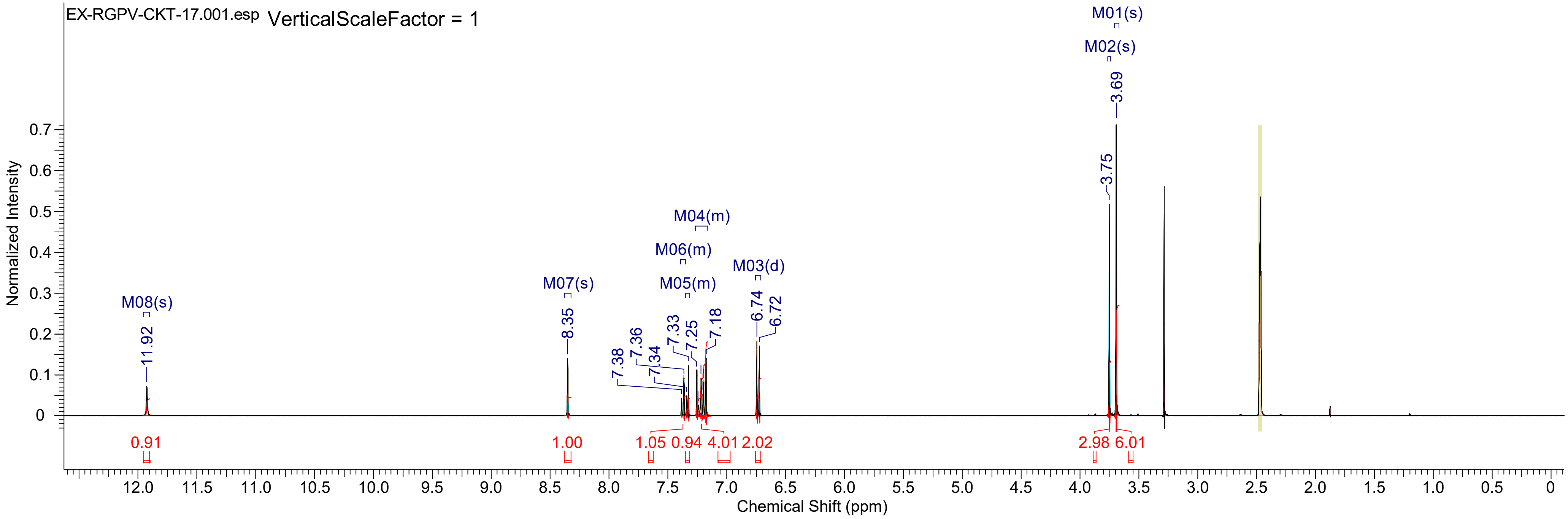

NMR SPECTRA OF CTR-40

23-11-2020 21:53:45

Formula $C_{21}H_{19}NO_4$	*FW* 349.3799

Acquisition Time (sec)	3.9977	*Comment*	PROTONRO DMSO /opt/topspin/nmrsu/EXTERNAL/MAR16 nmrsu			*Date*	14 Mar 2016 14:24:08
Date Stamp	14 Mar 2016 14:24:08						
File Name	E:\DELL COMPUTER DATA\D DRIVE JAN 2016\AMIT PROJECTS\IND-2 GRANT\NMR DATA\NEW NMR\NMR CKT_FEB2016\NMR fids-Spectra\NMR fids-Spectra\EX-RGPV-CKT-24\1\fid						
Frequency (MHz)	400.13	*Nucleus*	1H	*Number of Transients*	16	*Origin*	spect
Original Points Count	32768	*Owner*	nmrsu	*Points Count*	32768	*Pulse Sequence*	zg30
Receiver Gain	203.00	*SW(cyclical) (Hz)*	8196.72	*Solvent*	DMSO-d6	*Spectrum Offset (Hz)*	2384.6575
Spectrum Type	STANDARD	*Sweep Width (Hz)*	8196.47	*Temperature (degree C)*	25.260		

^{1}H NMR (400 MHz, DMSO-d_6) δ 11.94 (s, 1H), 8.33 (s, 1H), 8.00 (d, J = 15.76 Hz, 1H), 7.45 - 7.53 (m, 3H), 7.17 - 7.26 (m, 3H), 7.15 (d, J = 8.25 Hz, 1H), 7.02 (t, J = 7.38 Hz, 1H), 4.12 (q, J = 7.00 Hz, 2H), 3.77 (s, 3H), 1.31 (t, J = 7.00 Hz, 3H)

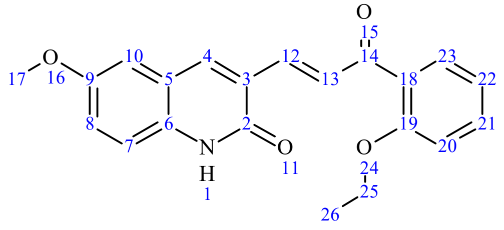

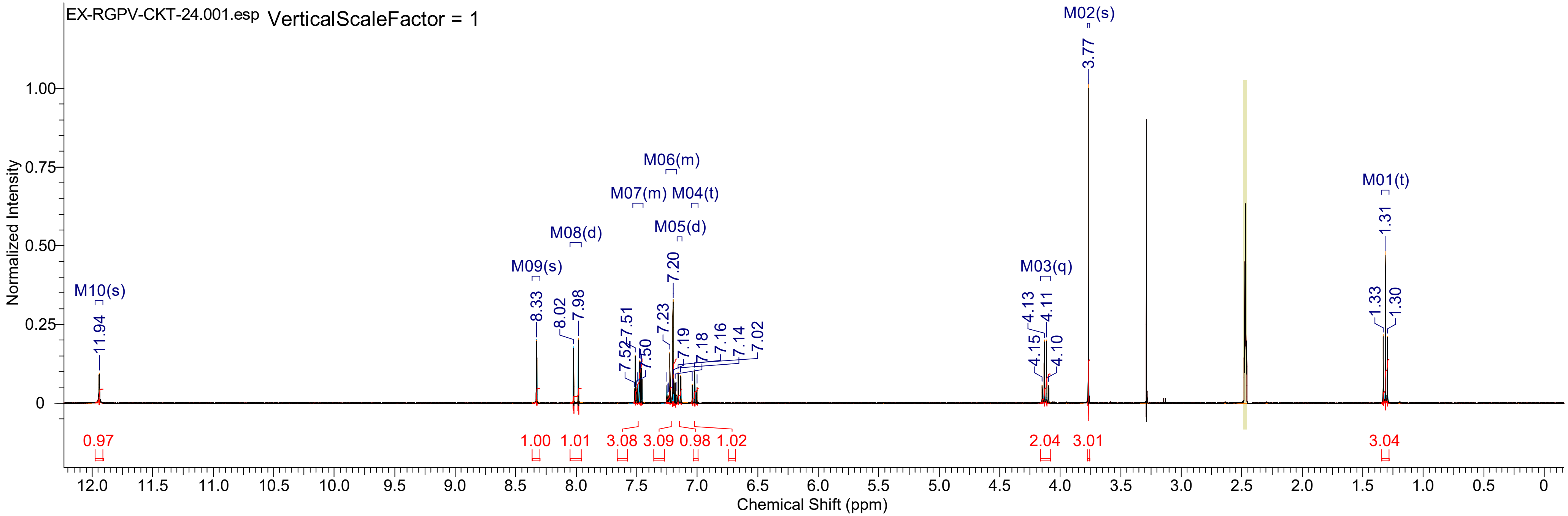

Supplement: Supplementary file 3 — Supplementary Information 3. [file 41598_2021_1058_MOESM3_ESM.pdf]
